# Unveiling the Evolutionary History of c*is‐*Andean *Alouatta* (Atelidae, Alouattinae) Through Mitochondrial Genomes

**DOI:** 10.1002/ajp.70043

**Published:** 2025-05-06

**Authors:** Cíntia Povill, Fabrícia F. Nascimento, Larissa Souza Arantes, Maximilian Driller, James Kieran Sullivan, Fernando Araujo Perini, Filipe Vieira Santos de Abreu, Ricardo Lourenço de Oliveira, Fabiano Rodrigues de Melo, Cecília Bueno, Camila J. Mazzoni, Cibele Rodrigues Bonvicino

**Affiliations:** ^1^ Pós‐graduação de Genética, Instituto de Biologia Universidade Federal do Rio de Janeiro Rio de Janeiro Brazil; ^2^ Department of Evolutionary Genetics Leibniz Institute for Zoo and Wildlife Research Berlin Germany; ^3^ Berlin Center for Genomics in Biodiversity Research Berlin Germany; ^4^ Department of Infectious Disease Epidemiology Imperial College London London UK; ^5^ Laboratório de Evolução de Mamíferos, Departamento de Zoologia Universidade Federal de Minas Gerais Belo Horizonte Minas Gerais Brazil; ^6^ LBPMR, Instituto Oswaldo Cruz, Fundação Oswaldo Cruz Rio de Janeiro Brazil; ^7^ Departamento de Engenharia Florestal Universidade Federal de Viçosa Viçosa Minas Gerais Brazil; ^8^ Universidade Veiga de Almeida, Centro de Estudos de Vertebrados Selvagens Rio de Janeiro Brazil

**Keywords:** genetic diversity, howler monkeys, mitochondrial DNA, Platyrrhini, species complex

## Abstract

*Alouatta*, a genus widely distributed throughout South and Central America, displays remarkable species diversity across various morphoclimatic domains. To clarify the ancestral distribution and its role in the radiation of *Alouatta*, our study employed time‐tree phylogenetic analyses to better understand the current distribution patterns of the *cis‐*Andean species. We generated 36 mitogenomes, including a species and representatives of populations not previously analyzed, to reconstruct a molecular‐dated tree, estimate genetic distance‐based analyses, and infer the ancestral distribution range of *Alouatta*. Our study suggests an initial split within the *Alouatta* during the Miocene, leading to the separation of the *cis‐*Andean and *trans*‐Andean clades. Through ancestral range reconstruction, we found that the most recent common ancestor of *Alouatta* was broadly distributed across South America. Within the *cis‐*Andean clade, two major splits were identified. One split revealed a close relationship between the Amazonia‐endemic species *A. seniculus* and *A. caraya*, a species adapted to open‐dry domains, with ancestral range in the Amazonia and dry‐open domains. In contrast, for the *A. guariba* and *A. belzebul* groups, which occur in Amazonia and the Atlantic Forest, the ancestral range included both domains. The diversification of the *Alouatta* was driven by two cladogenesis events. The formation of the extant species was primarily driven by founder events during the Pleistocene and involved long‐distance dispersal events with posterior population isolation. These events played a crucial role in the formation of new populations that underwent rapid divergence, resulting in distinct phylogenetic lineages. Our findings shed new light on the origins of *cis‐*Andean lineages of *Alouatta* across a broad geographic range, as well as the emergence of more recent taxa during the Pleistocene. This provides insights into their relationships, highlighting the crucial role of Pleistocene climatic changes and founder events in shaping the diversification and geographic distribution of extant species.

## Summary

- Mitochondrial DNA analysis suggests that founder‐event speciation played a key role in the divergence between cis‐Andean and trans‐Andean clades, as well as in the diversification of cis‐Andean lineages.
- Ancestral range reconstruction using mitochondrial genomes indicates that the most recent common ancestor of *Alouatta* had a widespread distribution across South America.
- Pleistocene climatic changes and riverine formations drove rapid divergence, shaping the geographic distribution and diversification of *Alouatta* species.

## Graphical abstract

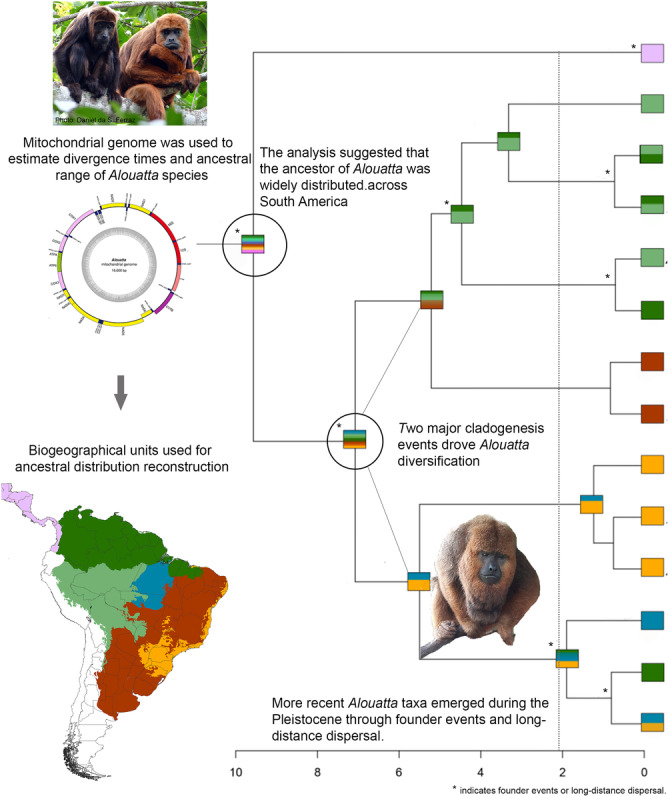

## 1 Introduction

**Figure 1:**
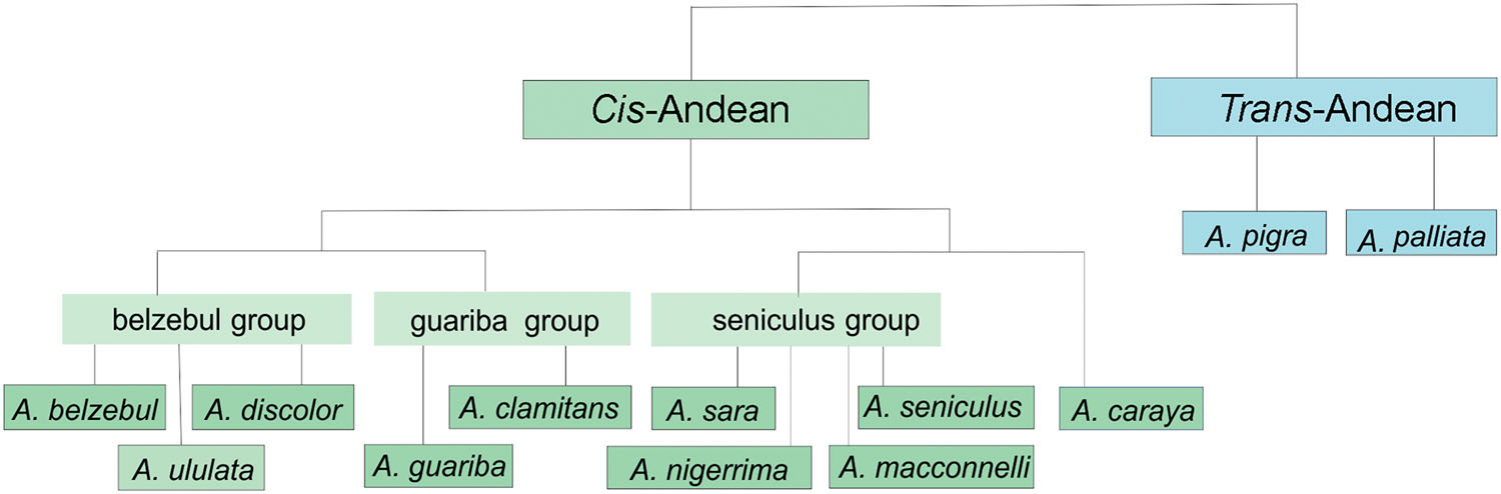
Division of species of the genus *Alouatta* into *cis* and *trans*‐Andean species. * *A. seniculus* includes *A.s. juara* and *A.s. puruensis*.

Platyrrhini is a primate group that comprises a large variety of species, inhabiting South and Central Americas through the south of Mexico. Within this group, the genus *Alouatta*, commonly known as howler monkeys, is distributed throughout the Americas from northern Argentina to the south of Mexico. There are 15 recognized species: 13 in the *cis‐*Andean group, distributed East of the Andes in South America, and two in the *trans‐*Andean group, distributed West of the Andes, including Mesoamerica (Cortés‐Ortiz et al. 2015; Gregorin 2006) (Figure 1). These species are further organized into groups based on taxonomy, morphology, and phylogenetic relationships (Cortés‐Ortiz et al. 2015; Gregorin 2006). The *A. seniculus* group comprises Amazonian species such as *A. seniculus* (including *A. s. puruensis* and *A. s. juara*), *A. sara* and *A. arctoidea* (Cortés‐Ortiz et al. 2015), *A. macconnelli* and *A. nigerrima* (Bonvicino et al. 2001). The *A. belzebul* group is composed of three species, *A. belzebul*, *A. discolor*, and *A. ululata* (Povill et al. 2022; Gregorin 2006). The *A. guariba* group includes *A. guariba* and *A. clamitans*, whose taxonomic status and distribution are still not well established, and the limit of their geographic distribution was recently reevaluated (Povill et al. 2023a). The phylogenetic relationships of *Alouatta caraya* are not established yet (Cortés‐Ortiz et al. 2003; Doyle et al. 2021; Do Nascimento et al. 2007). Therefore, studies focusing on the phylogenetic relationships within the *Alouatta* group are necessary to provide a more comprehensive understanding of its evolutionary history and clarify the relationships between different species.

Howler monkeys, particularly the *cis*‐Andean species, inhabit a diverse array of morphoclimatic environments throughout their range, exhibiting significant variations in pelage color, body shape, and the size and shape of the hyoid bone (Gregorin 2006). Despite their prominence in research, much of the phylogenetic inquiry into the *Alouatta* genus has relied on a single mitochondrial molecular marker, cytochrome *b*, typically limited to ≤ 1140 base pairs (de Mello Martins et al. 2011; Do Nascimento et al. 2007, 2008; Povill et al. 2022; Povill et al. 2023a). Furthermore, some investigations have focused solely on a subset of representative species, such as eight (Cortés‐Ortiz et al. 2003) or seven (Doyle et al. 2021) out of the 15 species. Unfortunately, these studies failed to establish a consensus on the evolutionary relationships among different *Alouatta* species. Understanding the phylogeny of howler monkeys and its alignment with historical biogeographic events is crucial for unraveling the evolutionary history of the *Alouatta* genus. By delving deeper into these connections, researchers can obtain valuable insights into the complex evolutionary processes that have shaped the diversity and distribution of these primates.

The mechanisms driving species diversity in the Amazon region are subject of ongoing debate. Two main explanations are usually proposed to explain species diversification in the region: 1) the refuge hypothesis, which proposes that climate changes and the expansion and contraction of the rainforest created isolation and differentiation of the species, and 2) the riverine barriers hypothesis, which proposes a large role of river dynamics as barriers to gene flow, leading to allopatric speciation (Ribas et al. 2012). Amazonian rivers, including the Amazonas, Negro, Tapajós, Madeira, Tocantins, and Xingu, are commonly referred to as important geographic barriers that promoted the speciation of several taxa, such as species of *Saimiri* (Lynch Alfaro et al. 2015) and *Alouatta* (Gregorin 2006). The rivers likely would have acted as barriers, facilitating the establishment of isolated populations that underwent rapid divergence. Additionally, climatic changes during the Pleistocene, characterized by glacial and interglacial periods, would have resulted in population contractions and expansions, respectively, which further contributed to the speciation processes. In contrast to previous results, a recent study (Janiak et al. 2022) showed that, particularly for *Alouatta*, Amazonian rivers may not have such a significant role in species divergence, documenting individuals of the same species on both sides of riverbanks. River characteristics, such as width and depth, as well as fluctuating water levels, may account for the intermittent presence or absence of individuals from different species on the same riverbank. However, it is important to consider the role of river formation processes in reducing population contact for some species, as well as the current configuration acting as a definitive barrier to gene flow.

While phylogenetic relationships within *Alouatta* are well‐established for most taxa, the range of the most recent common ancestor (MRCA) of the genus and extant species remains unknown, which is crucial information for understanding the evolution of the genus. Similarly, there is a lack of studies on the most recently diverged *Alouatta* taxa, representing a gap in the evolutionary history of this genus. To address these objectives, we estimated timetrees and carried out historical biogeographic analyses using novel and publicly available full mitochondrial genomes of *Alouatta* species. The mitochondrial genome possesses unique characteristics, including haploidy, a highly preserved gene structure, a fast mutation rate, high within‐cell copy numbers, and low costs when associated with high‐throughput sequencing methods, making it a powerful marker for analyzing phylogenetic relationships among closely related species (Amit Roy 2014).

## 2 Methods

### 2.1 Sampling and Research Ethics

The *Alouatta* samples used in this study were previously collected and used elsewhere (Povill et al. 2022, 2023a). The licenses were obtained at ICMBio (Chico Mendes Institute of Conservation of Biodiversity) and MMA (Ministry of the Environment) under SISBIO (System of Authorization and Information in Biodiversity) licenses: 54707–4 and 1187/2013 (Povill et al. 2023a); and 514/2014, and 474 73/2014—2nd correction—DILIC/IBAMA (Povill et al. 2022).

**Figure 2:**
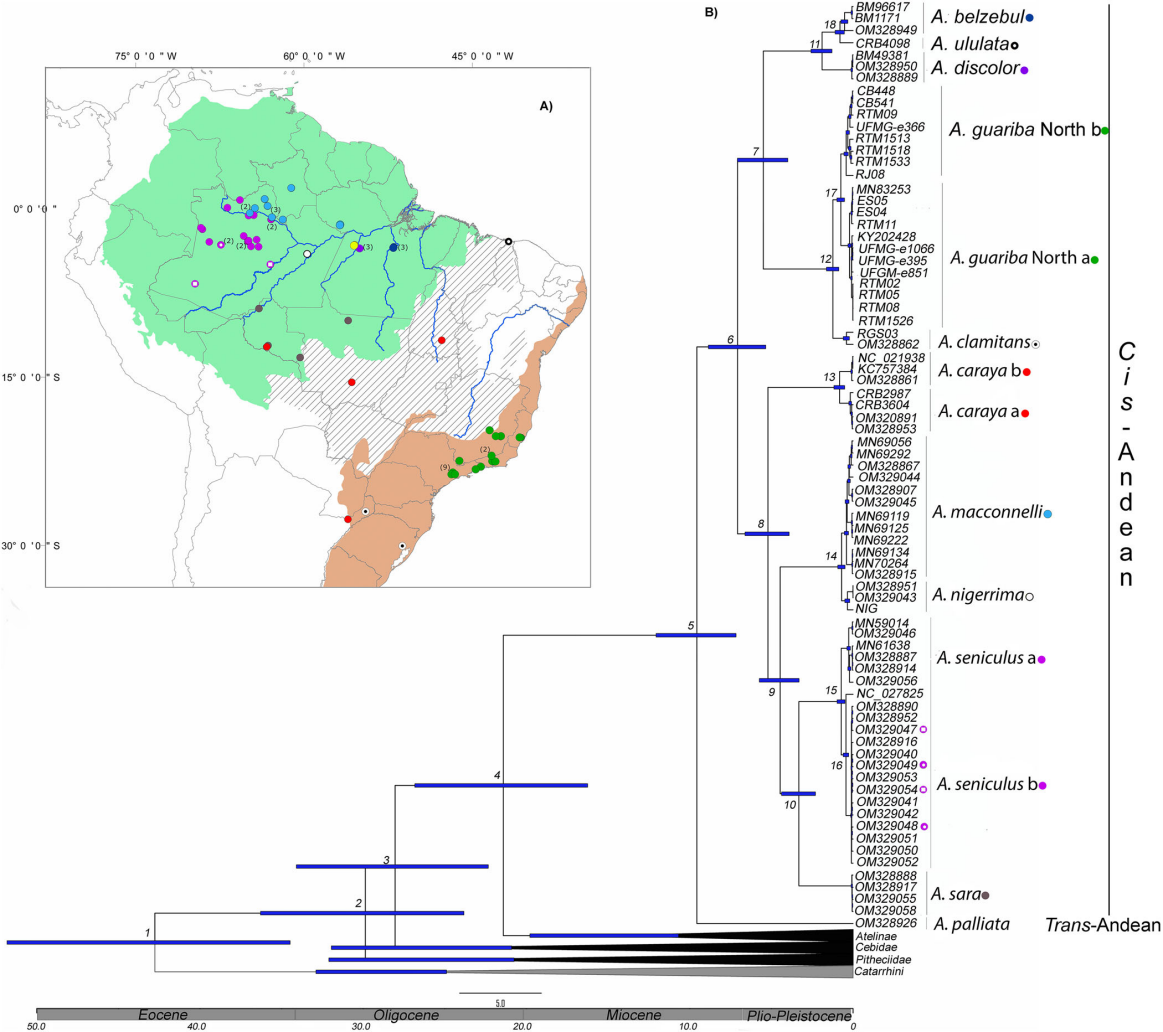
Map of the distribution of the *cis*‐Andean species (A) used in the analyses. The circles represent the collecting locations, and the colors represent different species. The number in parentheses represents the number of individuals used from the same location. The species of the *A. seniculus* group are represented by purple (*A. seniculus*) and shades of light pink (*A. s. puruensis* and *A. s. juara*); (B) the Bayesian time‐scaled tree inferred from mitogenomes, focusing on the *Alouatta* diversification. Bars indicate 95% highest posterior density (HPD) intervals of the age estimates in million years ago (Ma). The values close to the bars represent the number associated with Table 1 containing the node age estimates and credible interval. All nodes are supported with the highest value of posterior probability (PP = 1).

We analyzed 76 mitogenomes of *Alouatta* from different localities and species, including 36 sequenced here, being *A. belzebul* (*N* = 2), *A. discolor* (*N* = 1), *A. ululata* (*N* = 1), *A. caraya* (*N* = 2), *A. seniculus* (*N* = 2), *A. macconnelli* (*N* = 7), *A. nigerrima* (*N* = 1), and *A. guariba* group (*N* = 20) (Table S1). Additionally, we included 40 sequences obtained from GenBank, covering multiple *Alouatta* species, among which were individuals of *A. palliata*, and of the *A. seniculus* complex identified as *A. juara* and *A. puruensis* (Table 1; Figure 2a). As outgroups, we included representatives from families and genera of Platyrrhini and Catarrhini, including apes (Table S2).

**Table 1:** Samples of *Alouatta* used in the analysis with field or museum number (ID), GenBank accession number (GB), lineage, locality, and reference (REF). Ag = *A*. *guariba* group. BRA = Brazilian (BRA) states are Rio Grande do Sul (RS), São Paulo (SP), Minas Gerais (MG), Espírito Santo (ES), Rio de Janeiro (RJ), Tocantins (TO), Piauí (PI), Pará (PA), Amazonas (AM), Roraima (RR), and Mato Grosso (MT), ARG = Argentina. UFMG = Universidade Federal de Minas Gerais, PEFI = Parque Estadual das Fontes do Ipiranga. TS = This study, 1 = Janiak et al. (2022), 2 = Finstermeier et al. (2013), UN = Unpublished.

ID	GB	Lineage	Locality	Ref
RGS3	PV032489	*A. clamitans*	BRA: RS, Porto Alegre	TS
MACN52.41	OM328862	Ag South	ARG: Misiones	1
CB448	PV032510	Ag Northb	BRA: MG, Simão Pereira	TS
CB541	PV032512	Ag Northb	BRA: MG, Simão Pereira	TS
MN83253	PV032486	Ag Northa	BRA: RJ, Petrópolis	TS
RTM1513	PV032496	Ag Northa	BRA: SP, PEFI	TS
RTM08	PV032503	Ag Northa	BRA: SP, PEFI	TS
RTM05	PV032509	Ag Northa	BRA: SP, PEFI	TS
RTM1526	PV032511	Ag Northa	BRA: SP, PEFI	TS
RTM1518	PV032494	Ag Northb	BRA: SP, PEFI	TS
RTM1533	PV032490	Ag Northb	BRA: SP, PEFI	TS
UFMG‐e1066	PV032504	Ag Northa	BRA: MG, Rio Doce	TS
—	KY202428	Ag Northa	BRA: Unknown	UN
UFMG‐e366	PV032507	Ag Northb	BRA: RJ, Abre Campo	TS
UFMG‐e395	PV032508	Ag Northa	BRA: MG, Bom Jesus do Amparo	TS
UFMG‐e851	PV032497	Ag Northa	BRA: MG, Bom Repouso	TS
ES04	PV032495	Ag Northa	BRA: ES, Domingos Martins	TS
ES05	PV032479	Ag Northa	BRA: ES, Domingos Martins	TS
RJ08	PV032502	Ag Northb	BRA: RJ, Guapimirim	TS
RTM02	PV032500	Ag Northa	BRA: SP, PEFI	TS
RTM09	PV032506	Ag Northb	BRA: SP, PEFI	TS
RTM11	PV032480	Ag Northa	BRA: SP, PEFI	TS
BM96617	PV032499	*A. belzebul*	BRA: PA, Altamira, East Xingu River	TS
BM1171	PV032498	*A. belzebul*	BRA: PA, Altamira, East Xingu River	TS
BM52712	OM328949	*A. belzebul*	BRA: PA, Altamira, East Xingu River	1
BM49381	PV032482	*A. discolor*	BRA: PA, Vitória do Xingu, West Xingu River	TS
BM84342	OM328950	*A. discolor*	BRA: PA, Vitória do Xingu, West Xingu River	1
INPA7401	OM328889	*A. discolor*	BRA: PA	1
CRB4098	PV032492	*A. ululata*	BRA: PI, Delta do Rio Parnaíba	TS
MN 59014	PV032493	*A. seniculus*	BRA: AM, Barcelos	TS
MN 61638	PV032491	*A. seniculus*	BRA: AM, Barcelos	TS
JPB83	OM329056	*A. seniculus*	BRA: AM	1
IDSM03685	OM329052	*A. seniculus*	BRA: AM	1
IDSM03684	OM329053	*A. seniculus*	BRA: AM	1
IDSM03368	OM329050	*A. seniculus*	BRA: AM	1
IDSM03367	OM329051	*A. seniculus*	BRA: AM	1
INPA7530	OM329046	*A. seniculus*	BRA: AM	1
INPA7433	OM329042	*A. seniculus*	BRA: AM	1
INPA7430	OM329041	*A. seniculus*	BRA: AM	1
IDSM03694	OM329040	*A. seniculus*	BRA: AM	1
IDSM03683	OM328952	*A. seniculus*	BRA: AM	1
IDSM03369	OM328916	*A. seniculus*	BRA: AM	1
MN68607	OM328914	*A. seniculus*	BRA: AM	1
IDSM03682	OM328890	*A. seniculus*	BRA: AM	1
INPA8497	OM328887	*A. seniculus*	BRA: AM	1
—	NC027825	*A. seniculus*	Unknown	UN
IDSM00080	OM329049	*A. s. juara*	BRA: AM	1
IDSM00085	OM329048	*A. s. juara*	BRA: AM	1
UFRO577	OM329058	*A. sara*	BRA: RO	1
UFRO509	OM329055	*A. sara*	BRA: RO	1
IDSM03701	OM329054	*A. s. puruensis*	BRA: AM	1
INPA7499	OM329047	*A. s. puruensis*	BRA: AM	1
UFRO384	OM328917	*A. sara*	BRA: RO	1
UFMT4017	OM328888	*A. sara*	BRA: MT	1
MN69056	PV032485	*A. macconnelli*	BRA: AM, Barcelos	TS
MN 69292	PV032484	*A. macconnelli*	BRA: AM, Barcelos	TS
MN 69119	PV032488	*A. macconnelli*	BRA: AM, Barcelos	TS
MN 69222	PV032478	*A. macconnelli*	BRA: AM, Santa Isabel do Rio Negro	TS
MN 69125	PV032481	*A. macconnelli*	BRA: AM, Santa Isabel do Rio Negro	TS
MN 69134	PV032483	*A. macconnelli*	BRA: AM	TS
MN70264	PV032513	*A. macconnelli*	BRA: RR, Caracaraí	TS
INPA7520	OM329044	*A. macconnelli*	BRA: AM	1
INPA7475	OM329045	*A. macconnelli*	BRA: PA	1
INPA7545	OM328915	*A. macconnelli*	BRA: RR	1
INPA7471	OM328907	*A. macconnelli*	BRA: PA	1
INPA5715	OM328867	*A. macconnelli*	BRA: PA	1
NIG	PV032501	*A. nigerrima*	BRA: AM	TS
INPA7406	OM329043	*A. nigerrima*	BRA: PA	1
INPA7405	OM328951	*A. nigerrima*	BRA: PA	1
CRB2987	PV032487	*A. caraya*	BRA, MT, Chapada dos Guimarães	TS
CRB3604	PV032505	*A. caraya*	BRA: TO, Natividade	TS
UFROM573	OM328953	*A. caraya*	BRA: RO	1
UFROM576	OM328891	*A. caraya*	BRA: RO	1
AC10	OM328861	*A. caraya*	ARG: Corrientes	1
—	NC021938	*A. caraya*	Unknown	2
—	OM328926	*A. palliata*	Costa Rica	1

### 2.2 Library Preparation

DNA of liver or muscle tissue samples was isolated with Abcam Mitochondrial DNA Isolation Kit or following the phenol‐chloroform DNA extraction method (Sambrook and Russell 2001). We used two different library preparation methods, named here enrichment and long‐range PCR (LR‐PCR) (see Table S1 for samples‐specific information). The mitochondrial enrichment libraries were prepared for 17 out of 36 samples sequenced here following Abcam Mitochondrial DNA Isolation Kit for the DNA extraction. For the library preparation of the samples obtained from LR‐PCR, we carried out two LR‐PCRs using a set of primers designed for the samples of the *A. guariba* group (Table S1) in Primer3Plus (Untergasser et al. 2007). For the LR‐PCR, we used Platinum™ Taq DNA Polymerase High Fidelity and two sets of primers, the first set of primers was used to amplify approximately 8500 base pairs of the mitochondrial genome from ND4 to 16S genes (primers forward 5'‐GACCTATCCGTGAAGAGGCG‐3' and reverse 5'‐TAGGGTGGGGATTAGGGTGG‐3'), and the second set of primers was used to amplify approximately 9000 base pairs of the mitochondrial genome from 16S to ND4 (primer forward 5'‐AGCAACCCAACAACATCTCC‐3' and reserve 5'‐GGATTGCGCTGTTATCCCTA‐3'). The two sets of LR‐PCR produced overlapping regions, with approximately 271 base pairs between the forward primer of the first set and the reverse primer of the second set, and around 137 base pairs between the forward primer of the second set and the reverse primer of the first set.

For both methods, library preparation was performed using the Nextera XT DNA Library Preparation Kit (Illumina) following the manufacturer's recommendation. Libraries obtained with the enrichment method were indexed, pooled in equimolar ratios, and sequenced in a single lane on Hiseq. 2500 platform (Illumina), generating 2 ×150 bp paired‐end (PE) reads. Libraries obtained from the LR‐PCR method were sequenced in a single run on the Miseq platform (Illumina) generating 2 ×300 bp PE reads. Both methods were performed in different periods due to samples and resource availability.

### 2.3 Mitochondrial Genome Assembly Preprocessing

The sequencing quality was evaluated using the tools Fastqc version 0.11.9 (Andrews 2010) and Multiqc version 1.9 (Ewels et al. 2016). Paired‐end reads were filtered and trimmed by quality (Phred score > 32) and length (> 100 bp) using Trimmomatic version 0.38 (Bolger et al. 2014). PCR duplicates were removed using Picard version 2.21.8 (Picard Toolkit, 2019, Broad Institute, GitHub Repository. http://broadinstitute.github.io/picard/).

Trimmed reads of each sample were mapped against a curated reference mitogenome available in the NCBI RefSeq database (GenBank accession number NC_027825) and a mitogenome (GenBank accession number KY202428) available in the NCBI Nucleotide using Bowtie 2 (Langmead and Salzberg 2012), and visualized in Tablet (Milne et al. 2013). The mitochondrial genome assembly was performed with Novoplasty version 3.8.1 (Dierckxsens et al. 2017) using the mitogenomes available in the NCBI as references. Mauve version 2.4.0 (Darling et al. 2004) was used to reorganize the partitions of the 36 mitogenomes to the same order as the annotated reference sequences in the alignment. We used Mitos version 2 (Bernt et al. 2013) for annotation. Regions of the mitogenome were composed of 37 partitions (see illustration in Fig. S1; Greiner et al. 2019).

### 2.4 Sequence Alignments and Neighbor‐Net Analysis

We used 15 mitochondrial partitions, including 13 mitochondrial protein‐coding genes and rRNA 12S and 16S, to perform the phylogeny for 76 individuals (Table 1). We followed the taxonomic arrangement of Perelman et al. (2011) and included species of the families Atelidae, Cebidae, and Pitheciidae as well as Old World anthropoid species of the families Cercopithecidae, Hylobatidae and Hominidae as outgroups (Catarrhini). To generate the multiple sequence alignment, we used Mafft version 7 (Katoh and Standley 2013), and the alignments were manually checked with Mega version 11 (Tamura et al. 2021). Alignments were trimmed using TrimAl version 1.2 (Capella‐Gutiérrez et al. 2009) with the ‐gappyout command option to remove poorly aligned and uninformative sites. For every protein‐coding gene within each mitogenome (those sequenced in this study and those obtained from GenBank), we scrutinized the nucleotide sequences to identify any potential nuclear mitochondrial DNA pseudogenes (NUMTs). This involved translating the sequences into protein and evaluating their length as well as the occurrence of stop codons in incorrect positions. Nonetheless, we did not find any sequences suggestive of NUMTs. The phylogenetic positions deduced from the analyses were consistent with our expectations, suggesting that the presence of NUMTs did not impact our results.

Genetic distance‐based phylogenetic network (Neighbor‐Net) analysis was performed to calculate genetic distances between sequences (Bryant & Moulton 2004). We generated a genetic distance matrix for each protein‐coding gene using the Tamura‐3‐parameters model of nucleotide substitution (Tamura and Kumar 2002) in Mega version 11 and generated a matrix of inter and intraspecific genetic distance. We used POFAD version 1.03 (Joly and Bruneau 2006) to generate a matrix of distance with all genes combined, and SplitsTree version 4 (Huson 1998; Huson and Bryant 2006) to visualize the phylogenetic network of the combined‐genes matrix. We used the same genetic distance matrix to generate a heatmap based on sequence identity to visualize species' genetic distances using Biopython 1.81 (Cock et al. 2009) code DistanceCalculator(‘identity’). The “identity” is the model used to calculate the distance metric to compute the pairwise distances (Fig. S4).

### 2.5 Phylogenetics Analysis and Dating Trees

We estimated the Bayesian molecular clock dating of species divergence using over 13,000 nucleotides with 15 selected partitions, including 13 mitochondrial DNA protein‐coding genes and the 12S and 16S ribosomal RNA genes (Fig. S1). The analysis was performed using BEAST version 2.7.3 (Bouckaert et al. 2014). Clock and substitution models were linked for all partitions, with the clock model set to relaxed log‐normal and the substitution model set to GTR + I + G (Tavaré 1986; Yang 1994). This model, known for its parameter richness, accurately recovers phylogenetic relationships among genes and organisms (Abadi et al. 2019). The Markov chain Monte Carlo (MCMC) was run for 300,000,000 generations, sampling every 5,000 steps in two independent runs. We used the Bayesian Evolutionary Analysis Utility (BEAUti) package to prepare the BEAST input file, adding the prior distribution of tree node ages modeled with the Birth‐Death prior and a relaxed molecular clock that allows the substitution rates to vary across branches. The other parameters were kept as default. We used calibration points with uniform distribution based on a study of fossil calibrations for primate divergences (de Vries and Beck 2023) as follows: (1) the crown Anthropoidea at the lower and upper bound of 33.4 million years ago (Ma) and 56.03 Ma, the offset of 0.3 (de Vries and Beck 2023); (2) Catarrhini at the lower and upper bound of 24.7 and 33.4 Ma, the offset of 0.2; (3) Atelidae at the lower and upper bound of 12.5 and 34.9 Ma, offset of 0.2. We constrained the *Homo*‐*Pan* divergence at the lower and upper bound of 6.31 and 10 Ma, and the offset of 0.1 (Schrago and Seuánez 2019). For diagnostic MCMC output, we checked the effective sample size (ESS > 200) and the first 20% of trees were discarded as burn‐in. TreeAnnotator version 2.7.3, part of the BEAST package, was used to summarize the trees into a single maximum clade credibility tree, including the posterior probabilities of the nodes in the target tree and the highest posterior density interval (HPD) limits of the node heights.

We also estimated a dating tree using the least square dating (LSD2) method (To et al. 2016) of species divergence implemented in IQ‐TREE version 2.1.2 (Nguyen et al. 2015). This was carried out to compare with our Bayesian tree and to support our estimated dates. For the LSD2 analysis, we used the same data set, and the tree generated from the Bayesian analysis as input for the new calibration estimates. For the taxon divergence estimates, we included the following dating constraints: (1) Catarrhine at the lower and upper bound of 24.7 and 33.4 Ma; (2) Atelidae at the lower and upper bound of 12.5 and 34.9 Ma (de Vries and Beck 2023); and (3) *Homo*‐*Pan* divergence at the lower and upper bound of 6.31 and 10 Ma (Schrago and Seuánez 2019). The standard deviation of the lognormal relaxed clock was set to the default value of 0.2. We also estimated the maximum likelihood (ML) tree with IQ‐TREE for the 15 partitions concatenated. For the ML (Fig. S3), the species *Ateles belzebuth* (GenBank accession: NC 019800) and *Lagothrix lagotricha* (GenBank accession: NC 021951) were used as outgroups. We accounted for variation in substitution models and evolutionary rates among partitions, employing the *‐q* function, while also considering codon position. Branch support was assessed using the Shimodaira & Hasegawa‐like likelihood ratio test (SH‐aLRT; Guindon et al. 2010) and UFBoot2 (ultrafast bootstrap approximation; Hoang et al. 2018) with 3000 replicates. The final trees were visualized using Figtree version 1.3.1 (Rambaut 2010).

### 2.6 Analysis of Ancestral Reconstruction

We performed a biogeographical reconstruction with BioGeoBEARS version 1.1.3 (Matzke 2018) in R version 4.2.2 to estimate the range of ancestral distribution across time. We used a range of distribution slightly modified according to biogeographic areas and transition zones of Mesoamerica and South America (Morrone 2014). We defined the biogeographical regions as Northern Amazonia, South‐eastern Amazonia, South‐western Amazonia, Chaco (including Brazilian open vegetation ‐ Cerrado, Caatinga and Pantanal), *trans‐*Andean (North‐western South America + Mesoamerica), and Atlantic Forest. We tested three models, the dispersal‐extinction‐cladogenesis model (DECLIKE) and DECLIKE + j; the dispersal‐vicariance model (DIVALIKE) and DIVALIKE + j; and a model of discrete areas (BAYAREA) and BAYAREALIKE + j. The long‐distance dispersal parameter (j) was incorporated to account for founder‐event speciation (Matzke 2018). We performed the biogeographical analysis using the time‐scaled tree inferred with BEAST.

## 3 Results

### 3.1 Mitochondrial DNA Sequencing and Diversity

We sequenced and assembled 36 *Alouatta* complete mitogenomes [22 tRNA, 2 rRNA, the control region (d‐loop), and 13 protein‐coding genes] (Fig. S1; Table S1), including the first complete mitogenome generated for *A. ululata*. Our assembled mitogenomes, using mitochondrial enrichment and long‐range PCR showed differences in the number of reads. The LR‐PCR technique showed a larger number of reads and higher coverage of the assemblies compared to the samples processed with the enrichment method (Table S1).

We incorporated GenBank mitogenomes from individuals previously designated as *A. seniculus puruensis* (GenBank accession numbers: OM329058, OM329055, OM328917, OM328888). Nevertheless, following recent evidence from a study by Povill et al. (2023b), we reclassified these individuals as belonging to the species *A. sara* (Table 1).

### 3.2 Phylogenetic Relationships and Time Tree

The Bayesian molecular dating and LSD2 (Figure 2, Fig. S2) (Table 2) showed Platyrrhini divided into three main clades: Pitheciidae diverging first, separated from the clade grouping the families Cebidae and Atelidae. Within Atelidae, the *Alouatta* clade had two main divisions: one clade grouping *A. palliata*, representing the *trans*‐Andean species, and another grouping *cis‐*Andean species. The *cis‐*Andean clade was divided into two main clades, one grouping *A. nigerrima* and *A. macconnelli* as a sister clade to the monophyletic group containing the *A. seniculus* group (*A. seniculus*, *A. seniculus juara*, *A. seniculus puruensis*, *A. sara*) and *A. caraya*, and another clade grouping the *A. belzebul* group (*A. belzebul, A. ululata*, and *A. discolor*) as sister of the *A. guariba* group (*A. guariba* and *A. clamitans*). For Bayesian molecular clock dating all nodes were supported with the highest value of posterior probability (PP = 1), and for the ML phylogenetic analysis the supports were high, with values greater than 84.8 (aLRT) and 92 (bootstrap) (Fig. S3). Additionally, following the division within groups of species, such as *A. belzebul* and *A. guariba* groups, we further subdivided the *A. seniculus* clade. Specifically, we designated the *A. seniculus sensu stricto* as *A. seniculus* “a”, and delineated *A. seniculus* “b”, which encompasses individuals previously identified as *A. s. puruensis* and *A. s. juara*. Furthermore, we divided *A. caraya* into *A. caraya* “a” and *A. caraya* “b”.

**Table 2:** Estimates of divergence between Primates taxa obtained with the Bayesian (BY) and LSD2 clock dating, specifying the node age (N_age) estimates in millions of years ago (Ma), and the values of credible intervals (CI) representing the 95% highest posterior density (HPD). All nodes have the maximum value of posterior probability (PP = 1).

Node	Taxa	N_Age (LSD2)	N_Age (BY)	CI (BY)
1	Catarrhini‐Platyrrhini	45.96	42.77	34.48–51.82
2	Pitheciidae	26.12	29.88	23.84–36.3
3	Cebidae	26.24	28.05	22.34–34.1
4	Atelidae	21.42	21.42	16.26–26.85
5	*Alouatta* (*trans‐*and *cis‐*Andean)	9.57	9.57	7.17–12.07
6	*cis‐*Andean	7.07	7.07	5.35–8.86
7	*A. bezebul* group ‐ *A. guariba* group	5.5	5.5	3.99–7.08
8	*A. caraya* ‐ *A. macconnelli*/*A. nigerrima*/*A. seniculus* group	5.21	5.21	3.91–6.62
9	*A. macconnelli*/*A. nigerrima* ‐ *A. seniculus* group	4.47	4.27	3.31–5.74
10	*A. seniculus* ‐ *A. sara*	3.32	3.32	2.3–4.39
11	*A. belzebul* group	1.9	1.9	1.29–2.58
12	*A. guariba* group	1.24	1.24	0.87–1.63
13	*A. caraya*	0.83	0.83	0.51–1.17
14	*A. macconnelli* – *A. nigerrima*	0.7	0.7	0.49–0.94
15	*A. seniculus a ‐ A. seniculus b*	0.73	0.73	0.49–0.98
16	*A. seniculus* b	0.43	0.43	0.27–0.61
17	*A. guariba* North a ‐ *A. guariba* North b	0.75	0.75	0.52–0.98
18	*A. ululata* ‐ *A. belzebul*	0.8	0.8	0.53–1.1

The split between Catarrhini and Platyrrhini primates (Figure 2; Table 2) took place in the Eocene epoch. Within platyrrhines, the first split of the most recent common ancestral (MRCA) was dated at the end of the Eocene. The ancestor of *Alouatta* (*trans‐*and *cis‐*Andean extant species) diverged in the Miocene (9.57 Ma, Cl = 7.17–12.07), and the split between the *A. belzebul* and *A. guariba* groups occurred between the late Miocene and early Pliocene (5.50 Ma; Cl = 3.99–7.08); and the common ancestor of *A. nigerrima, A. macconnelli*, *A. seniculus, A. sara*, and *A. caraya* diverged in the early Pliocene at 5.21 Ma (Cl = 3.91–6.62). After that, the common ancestor of *A. macconnelli/A. nigerrima* and *A. seniculus/A. sara* rapidly diverged (4.47 Ma; Cl = 3.31–5.74). Within *A. belzebul* and *A guariba* groups, the divergence occurred in the Quaternary, during the Pleistocene, with recent splits. The split between *A. discolor* and *A. belzebul* occurred at 1.90 Ma (Cl = 1.29–2.58), and the most recent divergence was between *A. ululata* and *A. belzebul* at 0.80 Ma (Cl = 0.47–1.16). In the *A. guariba* group, the split between the North and South clades dated at 1.24 Ma (0.87–1.63) and clade North A from clade North B at 0.75 Ma (0.52–0.99). Within the *A. seniculus* group, we found a split of 0.73 Ma (Cl = 0.49–0.92) and 0.83 Ma within *A. caraya* (Cl = 0.51–1.17), and 0.70 Ma between *A. macconnelli* and *A. nigerrima* (Cl = 0.49–0.94). Bayesian dating and the LSD2 recovered similar times of divergences within *Alouatta* and between Platyrrhini families.

### 3.3 Phylogenetic Network Based on the Distance Matrix

**Figure 3:**
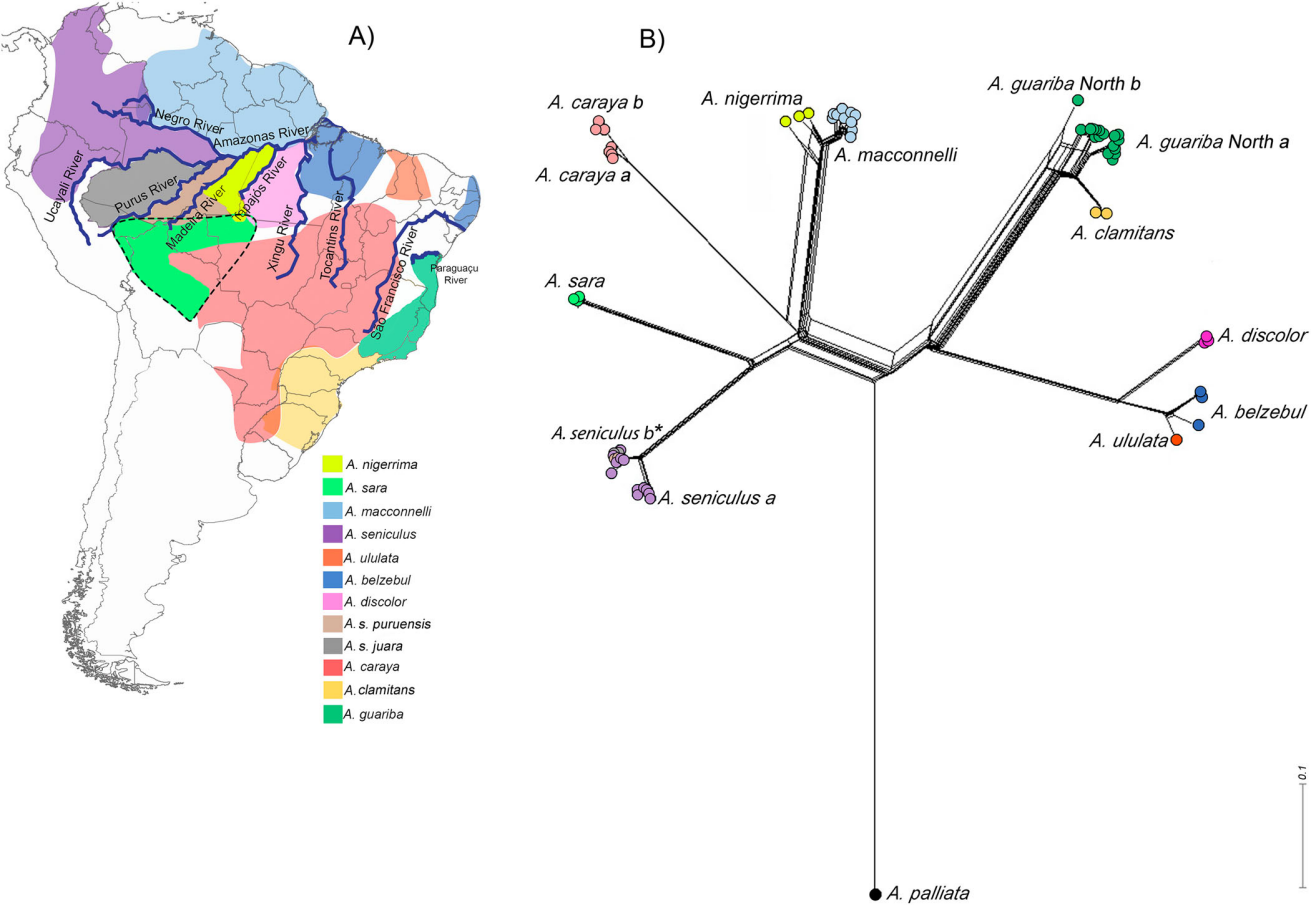
Distribution area of the *cis*‐Andean species of *Alouatta* and genetic distance between species. (A) Map of South America with the distribution of *Alouatta* species according to IUCN Red List, Povill et al. (2023a, 2023b); Cortés‐Ortiz et al. (2015), and Gregorin (2006); Dotted line represents the new distribution suggested for *A. sara* (Povill et al. 2023b). Each color represents one distinct species distribution, dashed lines represent the recently expanded distribution area of *A. sara*. (B) Neighbor‐net of *Alouatta* based on the genetic distance of 13 mitochondrial protein‐coding genes. The length of lines represents the standardized genetic distance among the lineages. Circles represent each sequence used and the colors are according to the map. *A. seniculus* ‘a’ refers to *A. seniculus* sensu stricto and *A. seniculus* ‘b’ refers to the group that includes *A. s. seniculus*, *A. s. puruensis*, *A. s. juara*. *A. guariba* North a and *A. guariba* North b refers to the lineages defined by Povill et al. (2023a).

The compiled distance matrix for each coding gene showed the genetic distances among the groups. *Alouatta palliata*, the only *trans*‐Andean species used in the analysis, exhibited the highest genetic distance value compared to all other *cis*‐Andean species. Within *cis*‐Andean species, the values of intraspecific genetic distances and interspecific genetic distances (Fig. S4) corroborate the relationships observed in the trees. The neighbor‐net, which also aligned with the species relationships, showed seven main splits with interspecific genetic distances between *cis‐*Andean species: (1) *A. nigerrima* and *A. macconnelli*, (2) *A. seniculus*, (3) *A. caraya*, (4) *A. belzebul* group ‐ *A. discolor, A. ululata*, and *A. belzebul*, (5) *A. guariba* group ‐ *A. guariba* (North a and b) and *A. clamitans*, and (6) *A. sara*, and (7) the *trans‐*Andean species *A. palliata* (Figure 3B). This analysis recovered *A. palliata* as the most divergent species, showing a higher genetic distance separating it from the remaining *cis‐*Andean species.

### 3.4 Ancestral Distribution Reconstruction

**Figure 4:**
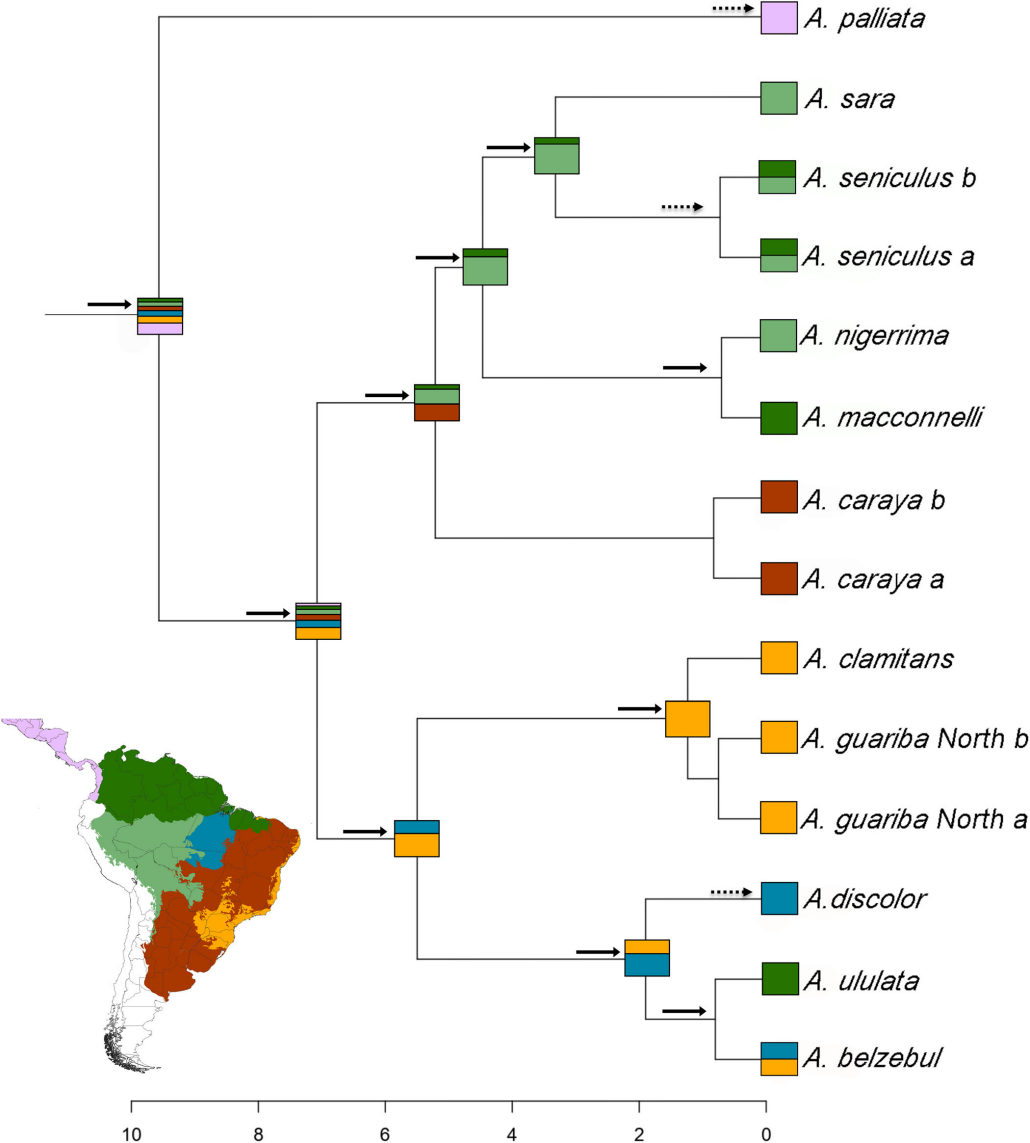
Historical biogeographical analysis of *Alouatta*. Squares at the nodes indicate the ancestral state/range probabilities of distribution. The areas were divided into six biogeographical units (Morrone 2014): *trans‐*Andean (lilac), south‐western Amazonia (light green), northern Amazon (dark green), south‐eastern Amazonia (blue), Atlantic Forest (yellow), and Chaco (including Brazilian Chaco ‐ Cerrado, Caatinga, and Pantanal) (brown). The squares at the tip represent the current distribution of each operational taxonomic unit. The numbers in the x‐axis represent the time million years ago. Dashed arrows indicate anagenetic dispersal events and solid arrows cladogenetic events.

The best‐fit model of historical biogeographical range, determined using the corrected Akaike information criterion (AICc) was BAYAREALIKE + j (see Figure 4; Table S3). Compared to the other models (Table S3), it exhibited the Log‐likelihood Score (LnL) of −28.54. The analysis suggests that the recent ancestral distribution of *Alouatta* comprised a large portion of South America. The model also showed that founder‐event or jump dispersal explained the ancestor range distributions better than the nested model. The ancestral range of the *A. guariba* and *A. belzebul* groups was in Amazonia and Atlantic Forest, while the ancestral range of the *A. seniculus* group and *A. caraya* was in Amazonia and dry‐open domains. The analysis revealed that the estimated ancestral range distributions of the ancestors have a large range of occupancy probabilities per area. This indicates the probability of each area at each node occupied over evolutionary time (Fig. S5).

## 4 Discussion

### 4.1 Evolutionary Origin of *Alouatta* Clades: Patterns of Divergence and Biogeographic Events

Our study supports the monophyly of the families Atelidae, Cebidae, and Pitheciidae, the latter as first offshoot. In the context of the genus *Alouatta*, our findings agree with previous studies based on mitochondrial markers (Janiak et al. 2022; de Mello Martins et al. 2011; Viana et al. 2015). We observed a close relationship between the *A. belzebul* and the *A. guariba* groups, while *A. caraya* is closely related to the clade comprising Amazonia‐endemic species of the *A. seniculus* group, *A. sara*, *A. macconnelli*, and *A. nigerrima*. It is important to note that our estimates of *Alouatta*'s evolutionary radiation differed from previous publications based on combined mitochondrial markers (Cortés‐Ortiz et al. 2003 – 6.8 Ma) and nuclear markers (Doyle et al. 2021 – 13.2 Ma; Kuderna et al. 2023 – 5.62 Ma). We attribute these variations to differences in calibration points, molecular markers, and sample compositions. Here we used a set of well‐calibrated priors (de Vries and Beck 2023) and well‐delimited sampling representing all *cis*‐Andean species, with exception of *A. arctoidea*. Regarding the timing of the first split within *Alouatta*, our study shows a new estimate, suggesting an approximately 9.57 Ma divergence, differing from previous studies (Cortés‐Ortiz et al. 2003; Doyle et al. 2021; Kuderna et al. 2023). However, all three studies consistently place the origin of the extant genus *Alouatta* in the Miocene epoch, specifically during the medium to late stages. This timeframe aligns with the development of the Amazon drainage basin, which is believed to have reached its current shape and size between 12.2 and 6.2 Ma (Méndez‐Camacho et al. 2021). These biogeographic events likely played a significant role in initiating the first divergences within the *cis‐*Andean clade of *Alouatta*. Additionally, during this period, the final stages of the northern Andean uplift were taking place (Hoorn et al. 2010). This uplift likely influenced the present‐day distribution patterns of various vertebrate species (Hoorn et al. 2010; Antonelli et al. 2010), including the divergence between *Alouatta* species in the *trans‐*Andean and *cis‐*Andean clades.

We conducted a thorough examination of the historical distribution of *Alouatta* MRCA and its extant species ancestors, shedding light on previously unexplored facets of its evolutionary history. Our findings from the dated‐historical biogeographical range analysis suggest that the MRCA of *Alouatta* had a broad distribution across South America during the Miocene epoch. While our analyses revealed that the distribution range of the *Alouatta* ancestor extended over both *trans*‐Andean (North‐western South America + Mesoamerica) and *cis*‐Andean regions, we posit that its primary distribution was centered in the North‐western and *cis*‐Andean regions of South America. This inference gained support from the emergence of the *cis*‐Andean ancestor soon after the split between *trans*‐Andean and *cis*‐Andean clades. Additionally, the formation of the Panama Isthmus, connecting South America with Central and North America, occurred roughly at 3 Ma, further reinforcing our proposal. However, we emphasize the limitations of our results due to the absence of sufficient representative samples from *trans*‐Andean species. Incorporating more data from *trans*‐Andean populations could refine the inferred biogeographic patterns, underscoring the need for future studies to address this sampling bias.

The analysis unveiled evidence of dispersal and subsequent colonization across diverse regions in South America within a relatively short time frame. This pattern aligns with the projected distribution area of the ancestral population. These events involved long‐distance dispersal, with posterior isolation of these populations, which may contribute to the narrowing of gene flow between populations. These mechanisms explained the two main cladogenesis events observed in the *Alouatta cis‐*Andean species. We propose that the initial divergence within the *cis‐*Andean species occurred approximately 5 Ma, resulting in the separation of the Amazonian‐endemic species and *A. caraya* from the *A. belzebul* and *A. guariba* groups, and facilitating the population dispersion. This divergence was concurrent with the expansion of savanna vegetation, encompassing the dry‐open Chaco, Cerrado, Caatinga, and Pantanal regions, collectively forming the arid diagonal of eastern South America (eSAAD). This expansion took place during the late Miocene period (11–5 Ma). The emergence of the eSAAD, along with the division of the Atlantic Forest from Amazonia, likely played a significant role in driving these divergent evolutionary paths (Ledo and Colli 2017; Pereira et al. 2022).

### 4.2 Paleoclimatic Fluctuations and Their Impact on Species Complex Formation and Relationships

Paleoclimatic fluctuations during the Pleistocene, characterized by glacial and interglacial periods, influenced the formation of recent species of plants and animals (Svenning et al. 2015). These climatic shifts played a pivotal role in shaping species complexes within *Alouatta*, driving its range shifts and diversification. These fluctuations likely triggered population reduction in *Alouatta* between approximately 1 Ma and 3 Ma (Kuderna et al. 2023), leading to population isolations and diversification within the genus, as suggested by our Bayesian molecular clock dating analysis. We suggest that intervals of branching events observed within the genus, with the divergence of *A. belzebul*, *A. guariba*, *A. seniculus* and *A. caraya* groups, are aligned with periods of these population reductions observed for *Alouatta* species (Kuderna et al. 2023). This may have facilitated the fragmentation and isolation of the remaining populations, ultimately resulting in genetic isolation and the emergence of new phylogenetic lineages, including extant species such as *A. macconnelli*/*A. nigerrima*, *A. seniculus*/*A. sara*, *A. belzebul*/*A. ululata*/*A. discolor*, and *A. guariba*/*A. clamitans*.

Despite the overlapping area observed between *A. sara* and *A. caraya* in Brazil and Bolivia (Povill et al. 2023b), our molecular Bayesian tree revealed a closer relationship between the Amazonian species *A. sara* and *A. seniculus*. The phylogenetic positioning of *A. caraya* in proximity to the Amazonian species agrees with previous studies using mitochondrial markers (Cortés‐Ortiz et al. 2003; Janiak et al. 2022) and 16 unlinked genes (Doyle et al. 2021), further supporting their close phylogenetic relationship. Our Bayesian dating analysis established that the divergence of *A. caraya* from the Amazonian endemic species occurred during the Pliocene epoch, a period when *A. caraya* ancestor likely exhibited adaptations to the eSAAD as suggested by the analysis of probability of occupancy per area, which illustrated the historical likelihood of species or lineages occupying specific regions. The eSAAD's formation, which commenced in the Miocene, served as a geographical barrier between the Atlantic Forest and Amazonia, influencing the separation of these two distinct morphoclimatic domains (Luebert 2021). It is plausible that the MRCA of *A. caraya* and the Amazonian endemic species resided in an area that was once encompassed by the Amazon and Atlantic Forest before the eSAAD's establishment.

Our results support the findings of previous research using mitochondrial markers (Cortés‐Ortiz et al. 2003; de Mello Martins et al. 2011; Bonvicino and Viana 2015; Povill et al. 2023a) indicating that the endemic Atlantic Forest *Alouatta guariba* group and the *A. belzebul* group share a common ancestor. This relationship suggests that there was a distribution of a common ancestor in both the eastern Amazon and the Atlantic Forest regions. The separation between the *A. guariba* and *A. belzebul* groups is currently influenced by the São Francisco river, which flows in the direction of the Atlantic Ocean, with *A. belzebul* populations located to the north and *A. guariba* populations to the south of this river (Gregorin 2006). The São Francisco river is one of the longest and the third largest river in Brazil (Kohler 2003) and has been identified as a geographic barrier influencing the speciation of various organisms, such as lizards (Passoni et al. 2008), rodents (Nascimento et al. 2011), and marsupials (Faria et al. 2013). During the Plio‐Pleistocene transition, the elevation of the Parnaiba river basin led to a change in the course of the São Francisco river, which shifted to a more northerly direction and encountered a transverse geological fault along Brazil's northeastern coast (Bruschi et al. 2019). This change in the São Francisco river course may have contributed to the complete isolation between the *A. guariba* and *A. belzebul* groups. However, the São Francisco river is not considered the main cause of the divergence between these species' groups. The connection between the North of the Atlantic Forest and Amazon biomes during various periods of the formation of dry areas (such as Caatinga, Cerrado, and Chaco) likely allowed for the movement of populations of *A. belzebul*, which have disjunct distributions in the Atlantic Forest and Amazon regions, facilitating gene flow (Batalha‐Filho et al. 2013; Povill et al. 2022). Furthermore, this connection was probably enabled by the expansion of *A. belzebul* after the change in the São Francisco river's course.

### 4.3 Pleistocene Epoch and Riverine Formations Explain the Species Splits

The Plio‐Pleistocene period was crucial for the diversification process of several platyrrhines, including *Sapajus*, Callicebinae, *Cacajao*, *Saimiri*, and Callithrichines (Boubli et al. 2015; Buckner et al. 2015; Byrne et al. 2018; Lima et al. 2017; Lynch Alfaro et al. 2015). This period also played a fundamental role in the speciation of *Alouatta* species, with the main process of cladogenesis occurring during this time. In the case of extant species, the Pleistocene epoch witnessed extensive diversification in various lineages, such as the *A. guariba* and *A. belzebul* groups. The riverine hypothesis suggests that geographic barriers created by Amazonian rivers led to the separation of organisms (Bonvicino and Weksler 2012; Marroig and Cerqueira 1997), including howler monkeys (Gregorin 2006). Pleistocene speciation events in several *Saimiri* species (Lynch Alfaro et al. 2015) and *Alouatta* species (Gregorin 2006) have been associated with Amazonian rivers, including the Negro, Tapajós, Madeira, Tocantins, and Xingu rivers. While the Tocantins river is a geographic barrier for some platyrrhine species (Lynch Alfaro et al. 2015), it does not seem to represent a barrier for *A. belzebul* populations, which occur on both riverbanks without geographic and genetic structuring. However, the Xingu river appears to have played a role in the divergence between *A. discolor* and *A. belzebul* (Povill et al. 2022).

Recent research challenges the traditional notion that Amazonian rivers restricted gene flow between *Alouatta* species (Janiak et al. 2022). However, we do not fully agree with this interpretation. In general, *Alouatta* species are well‐defined, with their distributions largely delimited by rivers. Additionally, some species, such as *A. belzebul* and *A. discolor*, show clear separation by Xingu river (Povill et al. 2022). Another factor to consider is the continuous reduction of river levels in the Amazonia region, facilitating the movement of individuals across rivers, although the observation of individuals on the opposite riverbank from their main population range does not necessarily mean they permanently inhabit these areas. This situation highlights the need for further investigation into the dynamic interactions between environmental changes, gene flow, and species distributions in *Alouatta*.

We propose that during the formation of the drainage systems, Amazonian rivers may have limited or reduced gene flow among several extant *Alouatta* species. The ongoing process of drainage formation has led to various changes in river size, shape, and direction, impacting species distribution and gene flow. Nevertheless, large rivers like Negro, Amazonas, Xingu, and Madeira, in their present configurations, act as geographic barriers, hindering and reducing gene flow between populations of Amazonian endemic *Alouatta* species but not totally avoiding the contact between populations. The establishment of these rivers associated with the Pleistocene events favored genetic differentiation between populations.

The presence of individuals from different species at the same riverbank of huge rivers could be the result of crossings facilitated by the presence of islands, creeks, meander formations, and variations in water levels during flood and drought seasons. Although *Alouatta* species tend to not move long distances, they are capable swimmers (Aguiar et al. 2007), but it remains unclear whether they can survive long‐distance swims across large rivers. Additionally, their presence in new localities may also result from human‐mediated transportation, as they are sometimes kept and relocated as pets (Duarte‐Quiroga and Estrada 2003).

### 4.4 Genetic Distances Among Species

The neighbor‐net analysis revealed genetic distances with equal arrangements among the branches seen in the phylogenetic tree*: A. belzebul* + *A. discolor* + *A. ululata* (*A. belzebul* group), *A. guariba* + *A. clamitans* (*A. guariba* group), *A. seniculus*, *A. sara*, *A. macconnelli* + *A. nigerrima*, *A. caraya*, and *A. palliata*. This finding aligns to the *Alouatta* splits and species relationships observed in the phylogenetic analysis, supporting the process of rapid diversification of *Alouatta* across various morphoclimatic domains such as Amazonia, dry‐open (Chaco, Cerrado, Caatinga and Pantanal), and Atlantic Forest. It also indicates a low genetic distance between closely related species within the *A. guariba* and *A. belzebul* groups and between *A. macconnelli* and *A. nigerrima*. The estimate of genetic distances analyses further supports the hypothesis of the time‐scaled tree that geological (such as river formations) and climatic events (such as glacial and interglacial periods) led to the simultaneous divergence of the *cis‐*Andean clade during the late Miocene. It highlights that the *A. guariba* and *A. belzebul* groups are genetically closer to each other and are separated by a significant distance from the other Amazonian and dry‐open domains adapted species.

Our analysis revealed that the date and branch lengths within the clades of *A. seniculus* species closely resemble those found within other species complexes, such as the *A. guariba* and *A. belzebul* groups. Considering karyotype evidence, obtained from individuals of *A. seniculus* (2*n* = 44) collected by C.R. Bonvicino in the Rio Negro basin, and patterns observed within other species groups with recent splits, we opted to divide the *A. seniculus* group clade. The *A. seniculus* “a” represents the *A. seniculus sensu stricto* while *A. seniculus* “b” represents a clade containing individuals previously identified as *A. s. juara* and *A. s. puruensis*. However, the lack of molecular and taxonomic studies on the *A. seniculus* group constraints further discussion on the distribution and taxonomic status of *A. s. juara* and *A. s. puruensis*. Both, *A. s. puruensis* and *A. s. juara*, are not genetically well characterized as distinct evolving lineage. Furthermore, due to the recent divergence between certain species, these primates can occasionally interbreed, and their mitochondrial DNA can exhibit ancestral polymorphism, which may complicate the understanding of geographic distribution boundaries. As a result, we propose that future studies reassess their taxonomic status and classification.

## 5 Conclusions

We presented new evidence supporting the divergence between the *trans‐*Andean and *cis‐*Andean species of *Alouatta*, as well as the range/state of distribution of the most recent common ancestor of the genus and its extant species. Our findings suggest that the geographic origin of the MRCA of both *trans‐*Andean and *cis‐*Andean species had a broad distribution primarily in South America. The *cis‐*Andean species dispersed and colonized other regions in a short time. The Miocene epoch played a significant role in the cladogenesis of *Alouatta*, marked by the split between the *trans‐*Andean and *cis‐*Andean clades, as well as two subsequent splits within the *cis‐*Andean clade caused by founder events. These events resulted in the emergence of Amazonia‐endemic species, dry‐open adapted species, Atlantic Forest endemic species, and species adapted to both Amazonia and Atlantic Forests. Notably, Pleistocene climatic changes and riverine basin formations created favorable conditions for population reduction, triggering rapid divergence between species, leading to speciation and the establishment of the extant species. Our study significantly contributes to our understanding of the evolutionary history of *Alouatta*, shedding new light on its phylogenetic relationships and the factors that have shaped its present‐day distribution across South America's diverse morphoclimatic domains.

## Author Contributions

**Cíntia Povill:** conceptualization (lead), data curation (equal), formal analysis (lead), investigation (lead), methodology (lead), software (lead), validation (equal), visualization (equal), writing – original draft (lead), writing – review and editing (lead). **Fabrícia F. Nascimento:** funding acquisition (equal), writing – original draft (supporting), writing – review and editing (equal). **Larissa Souza Arantes:** formal analysis (supporting), writing – original draft (equal), writing – review and editing (equal). **Maximilian Driller:** formal analysis (supporting), writing – original draft (equal), writing – review and editing (equal). **Fernando Araujo Perini:** data curation (equal), writing – review and editing (equal). **Filipe Vieira Santos de Abreu:** data curation (equal), writing – review and editing (equal). **Ricardo Lourenço de Oliveira:** data curation (equal), writing – review and editing (equal). **Fabiano Rodrigues de Melo:** data curation (equal), writing – review and editing (equal). **Cecília Bueno:** data curation (equal), writing – review and editing (equal). **Camila J. Mazzoni:** resources (equal), writing – review and editing (equal). **Cibele Rodrigues Bonvicino:** conceptualization (equal), data curation (equal), funding acquisition (lead), investigation (equal), project administration (lead), resources (equal), supervision (equal), writing – original draft (equal), writing – review and editing (equal).

## Conflicts of Interest

The authors declare no conflicts of interest.

## Supporting information

Supporting information MinorReview 31032025.

## Data Availability

The mitochondrial genomes generated and analyzed in this study have been deposited in the GenBank repository under accession numbers PV032478‐PV032513.
