# Supplementary material for: Unveiling the Evolutionary History of cis‐Andean Alouatta (Atelidae, Alouattinae) Through Mitochondrial Genomes
Source: Am J Primatol. 2025 May 6;87(5):e70043. doi: 10.1002/ajp.70043 (PMC12053967; doi:10.1002/ajp.70043)
Supplement: Supplementary file 1 — Supporting information MinorReview 31032025. [file AJP-87-e70043-s001.docx]

Unveiling the evolutionary history of c*is-*Andean *Alouatta* (Atelidae, Alouattinae) through mitochondrial genomes

Cíntia Povill, Fabrícia F. Nascimento, Larissa Souza Arantes, Maximilian Driller; Fernando Araujo Perini, Filipe Vieira Santos de Abreu, Ricardo Lourenço de Oliveira, Fabiano Rodrigues de Melo, Cecília Bueno, Camila J. Mazzoni, Cibele Rodrigues Bonvicino

**Supplementary Material**

Table S1. Samples sequenced here and used in the mitochondrial analyses, containing the Samples identification, GenBank accession number (GB), species names, number of reads used in the mitochondrial genome assembly, and its consensus length per individual. *=Long range PCR, ^1^= enrichment method.

| Sample | GB | Species | # of reads | Consensus Length |
| --- | --- | --- | --- | --- |
| BM96617^1^ | PV032499 | *A. belzebul* | 4,188 | 16,584 |
| BM49381^1^ | PV032482 | *A. discolor* | 49,376 | 16,587 |
| BM1171^1^ | PV032498 | *A. belzebul* | 5,352 | 16,584 |
| CRB4098^1^ | PV032492 | *A. ululata* | 5,408 | 16,583 |
| CRB2987^1^ | PV032487 | *A. caraya* | 15,006 | 16,519 |
| CRB3604^1^ | PV032505 | *A. caraya* | 14,114 | 16,520 |
| RTM1513* | PV032496 | *A. guariba guariba* | 250,536 | 16,590 |
| RTM1518* | PV032494 | *A. guariba guariba* | 296,578 | 16,590 |
| RTM1526* | PV032511 | *A. guariba guariba* | 251,284 | 16,590 |
| RTM1533* | PV032490 | *A. guariba guariba* | 315,962 | 16,590 |
| CB618^1^ | PV032486 | *A. guariba guariba* | 108,628 | 16,589 |
| CB448* | PV032510 | *A. guariba guariba* | 297,460 | 16,591 |
| CB541^1^ | PV032512 | *A. guariba guariba* | 15,668 | 16,591 |
| UFMG-e1066* | PV032504 | *A. guariba guariba* | 184,938 | 16,590 |
| UFMG-e366* | PV032507 | *A. guariba guariba* | 258,054 | 16,591 |
| UFMG-e395* | PV032508 | *A. guariba guariba* | 183,612 | 16,590 |
| UFMG-e851* | PV032497 | *A. guariba guariba* | 314,826 | 16,589 |
| ES05* | PV032479 | *A. guariba guariba* | 293,138 | 16,589 |
| ES04* | PV032495 | *A. guariba guariba* | 266,736 | 16,589 |
| RJ08* | PV032502 | *A. guariba guariba* | 200,752 | 16,590 |
| RTM02* | PV032500 | *A. guariba guariba* | 260,044 | 16,590 |
| RTM05* | PV032509 | *A. guariba guariba* | 21,299 | 16,590 |
| RTM08* | PV032503 | *A. guariba guariba* | 271,432 | 16,590 |
| RTM09* | PV032506 | *A. guariba guariba* | 301,216 | 16,590 |
| RTM11* | PV032480 | *A. guariba guariba* | 274,578 | 16,589 |
| RGS3* | PV032489 | *A. guariba clamitans* | 327,036 | 16,600 |
| MN59014^1^ | PV032493 | *A. seniculus* | 17,600 | 16,579 |
| MN61638^1^ | PV032491 | *A. seniculus* | 28,140 | 16,569 |
| MN70264^1^ | PV032513 | *A. macconnelli* | 25,194 | 16,553 |
| MN69056^1^ | PV032485 | *A. macconnelli* | 7,124 | 16,563 |
| MN69119^1^ | PV032488 | *A. macconnelli* | 22,546 | 16,620 |
| MN69125^1^ | PV032481 | *A. macconnelli* | 26,720 | 16,552 |
| MN69134^1^ | PV032483 | *A. macconnelli* | 5,714 | 16,561 |
| MN69292^1^ | PV032484 | *A. macconnelli* | 9,904 | 16,566 |
| MN69222^1^ | PV032478 | *A. macconnelli* | 25,442 | 16,560 |
| NIG^1^ | PV032501 | *A. nigerrima* | 33,818 | 16,570 |

Table S2. List of samples for all Platyrrhini and Catarrhini species, including apes, used as outgroups in the Bayesian dating tree analysis. The table includes GenBank accession numbers (GB), species names (as designated in the GenBank), and corresponding references.

| GB | Species | Reference |
| --- | --- | --- |
| NC_019800 | *Ateles belzebuth* | Hodgson et al. 2009 |
| OM328892 | *Ateles chamek* | Janiak et al. 2022 |
| OM328896 | *Ateles marginatus* | Janiak et al. 2022 |
| OM328927 | *Ateles geoffroyi* | Janiak et al. 2022 |
| OM328958 | *Ateles paniscus* | Janiak et al. 2022 |
| NC_021951 | *Lagothrix lagotricha* | Finstermeier et al. 2013 |
| NC_021939 | *Aotus azarai* | Finstermeier et al. 2013 |
| NC_019799 | *Aotus lemurinus* | Hodgson et al. 2009 |
| NC_018116 | *Aotus nancymaae* | Babb et al. 2011 |
| NC_002763 | *Cebus albifrons* | Arnason et al. 2000 |
| OM328897 | *Cebus olivaceus* | Janiak et al. 2022 |
| OM328874 | *Cebus unicolor* | Janiak et al. 2022 |
| NC_021961 | *Sapajus xanthosternos* | Finstermeier et al. 2013 |
| NC_050883 | *Sapajus flavius* | Hao et al. 2019 |
| OM328872 | *Sapajus apella macrocephalus* | Finstermeier et al. 2013 |
| OM329025 | *Sapajus apella* | Finstermeier et al. 2013 |
| OM328873 | *Saimiri ustus* | Finstermeier et al. 2013 |
| OM329003 | *Saimiri cassiquiarensi* | Finstermeier et al. 2013 |
| OM329012 | *Saimiri macrodon* | Finstermeier et al. 2013 |
| NC_023211 | *Saimiri oerstedii* | Chiou et al. 2011 |
| NC_021966 | *Saimiri boliviensis* | Finstermeier et al. 2013 |
| NC_012775 | *Saimiri sciureus* | Matsui et al. 2009 |
| NC_021941 | *Callithrix geoffroyi* | Finstermeier et al. 2013 |
| NC_025586 | *Callithrix jacchus* | Wang et al. 2014 |
| NC_030788 | *Callithrix penicillata* | Unpublished |
| NC_027658 | *Callithrix kuhlii* | Zhang et al. 2015 |
| NC_050682 | *Callithrix aurita* | Malukiewicz et al. 2021 |
| NC_021942 | *Cebuella pygmaea* | Finstermeier et al. 2013 |
| OM328875 | *Cebuella niveiventris* | Janiak et al. 2022 |
| OM328864 | *Mico humilis* | Janiak et al. 2022 |
| NC_064174 | *Mico humeralifer* | Janiak et al. 2022 |
| NC_064175 | *Mico argentatus* | Janiak et al. 2022 |
| NC_024628 | *Callimico goeldii* | Menezes et al. 2003 |
| NC_037878 | *Leontophitecus chrysopygus* | de Freitas et al. 2018 |
| NC_021952 | *Leontophitecus rosalia* | Finstermeier et al. 2013 |
| OM328871 | *Saguinus inustus* | Janiak et al. 2022 |
| OM328998 | *Saguinus labiatus rufiventer* | Janiak et al. 2022 |
| OM329001 | *Saguinus mystax* | Janiak et al. 2022 |
| OM328925 | *Saguinus geoffroyi* | Janiak et al. 2022 |
| NC_021960 | *Saguinus oedipus* | Finstermeier et al. 2013 |
| OM328996 | *Saguinus bicolor* | Janiak et al. 2022 |
| OM328999 | *Saguinus midas* | Janiak et al. 2022 |
| OM328879 | *Leontocebus nigricolli* | Janiak et al. 2022 |
| OM328797 | *Leontocebus fuscicolli* | Janiak et al. 2022 |
| NC_021946 | *Chiropotes albinaus* | Finstermeier et al. 2013 |
| NC_024629 | *Chiropotes israelita* | Menezes et al. 2003 |
| OM328899 | *Chiropotes chiropotes* | Janiak et al. 2022 |
| NC_021967 | *Cacajao calvus* | Finstermeier et al. 2013 |
| OM328880 | *Cacjao melanocephalus* | Janiak et al. 2022 |
| OM328881 | *Cacajao ayresi* | Janiak et al. 2022 |
| OM328910 | *Cacajao hosomi* | Janiak et al. 2022 |
| OM328877 | *Pithecia vanzolinii* | Janiak et al. 2022 |
| OM328903 | *Pithecia hirsuta* | Janiak et al. 2022 |
| OM328901 | *Pithecia pissinattii* | Janiak et al. 2022 |
| OM328904 | *Pithecia mittimeieri* | Janiak et al. 2022 |
| OM328905 | *Pithecia albicans* | Janiak et al. 2022 |
| OM328902 | *Pithecia chrysocephala* | Janiak et al. 2022 |
| NC_021965 | *Plecturocebus cupreus* | Finstermeier et al. 2013 |
| OM328876 | *Plecturocebus cupreus* | Janiak et al. 2022 |
| OM328930 | *Plecturocebus brunneus* | Janiak et al. 2022 |
| OM328931 | *Plecturocebus caligatus* | Janiak et al. 2022 |
| OM328933 | *Plecturocebus dubius* | Janiak et al. 2022 |
| OM328929 | *Plecturocebus bernhardi* | Janiak et al. 2022 |
| OM328934 | *Plecturocebus grovesi* | Janiak et al. 2022 |
| OM328994 | *Plecturocebus moloch* | Janiak et al. 2022 |
| OM328932 | *Plecturocebus cinerascens* | Janiak et al. 2022 |
| OM328936 | *Plecturocebus miltoni* | Janiak et al. 2022 |
| OM328935 | *Plecturocebus hoffmanni* | Janiak et al. 2022 |
| NC_019801 | *Plecturocebus donacophilus* | Hodgson et al. 2009 |
| NC_024630 | *Cheracebus lugens* | Menezes et al. 2003 |
| OM328863 | *Cheracebus lugens* | Janiak et al. 2022 |
| OM328940 | *Cheracebus regulus* | Janiak et al. 2022 |
| OM328941 | *Cheracebus torquatus* | Janiak et al. 2022 |
| OM328944 | *Cheracebus lucifer* | Janiak et al. 2022 |
| NC_001645 | *Gorilla gorila* | Horai et al. 1995 |
| NC_012920 | *Homo sapiens* | Andrews et al. 1999 |
| NC_001643 | *Pan troglodytes* | Horai et al. 1995 |
| NC_001646 | *Pongo pygmaeus* | Horai et al. 1995 |
| NC_014045 | *Hylobates pileatus* | Matsudaira et al. 2010 |
| NC_014047 | *Symphalangus syndactylus* | Matsudaira et al. 2010 |
| NC_018753 | *Nomascus gabriellae* | Chan et al. 2010 |
| NC_020009 | *Papio papio* | Zinner et al. 2013 |
| NC_024933 | *Chlorocebus cynosuros* | Wang et al. 2014 |
| NC_006901 | *Colobus guereza* | Raaum et al. 2005 |

Table S3. Estimated models that better explain the ancestral range of *Alouatta,* including [Log-likelihood Score](https://www.researchgate.net/figure/ncrease-in-Log-likelihood-Score-lnL-as-a-Result-of-Branch-Length-Optimization_tbl1_11139522) (LnL), number of parameters (Numparams), Akaike information criterion (AICc) and Akaike information criterion with correction weight (AICc_wt). The anagenetic processes “d” and “e” represent dispersal and extinction, respectively, and “j” represents a cladogenetic process of founder event or jump dispersal event.

| **Model** | **LnL** | **numparams** | **d** | **e** | **j** | **AICc** | **AICc_wt** |
| --- | --- | --- | --- | --- | --- | --- | --- |
| DEC | -34.26 | 2 | 0.063 | 0.064 | 0 | 73.60 | 0.014 |
| DEC+J | -31.39 | 3 | 0.016 | 1.0e-12 | 0.087 | 71.17 | 0.049 |
| DIVALIKE | -32.88 | 2 | 0.044 | 0.015 | 0 | 70.85 | 0.057 |
| DIVALIKE+J | -31.67 | 3 | 0.023 | 1.0e-12 | 0.043 | 71.73 | 0.037 |
| BAYAREALIKE | -35.78 | 2 | 0.075 | 0.16 | 0 | 76.65 | 0.0031 |
| BAYAREALIKE+J | -28.54 | 3 | 0.011 | 0.029 | 0.077 | 65.48 | 0.84 |


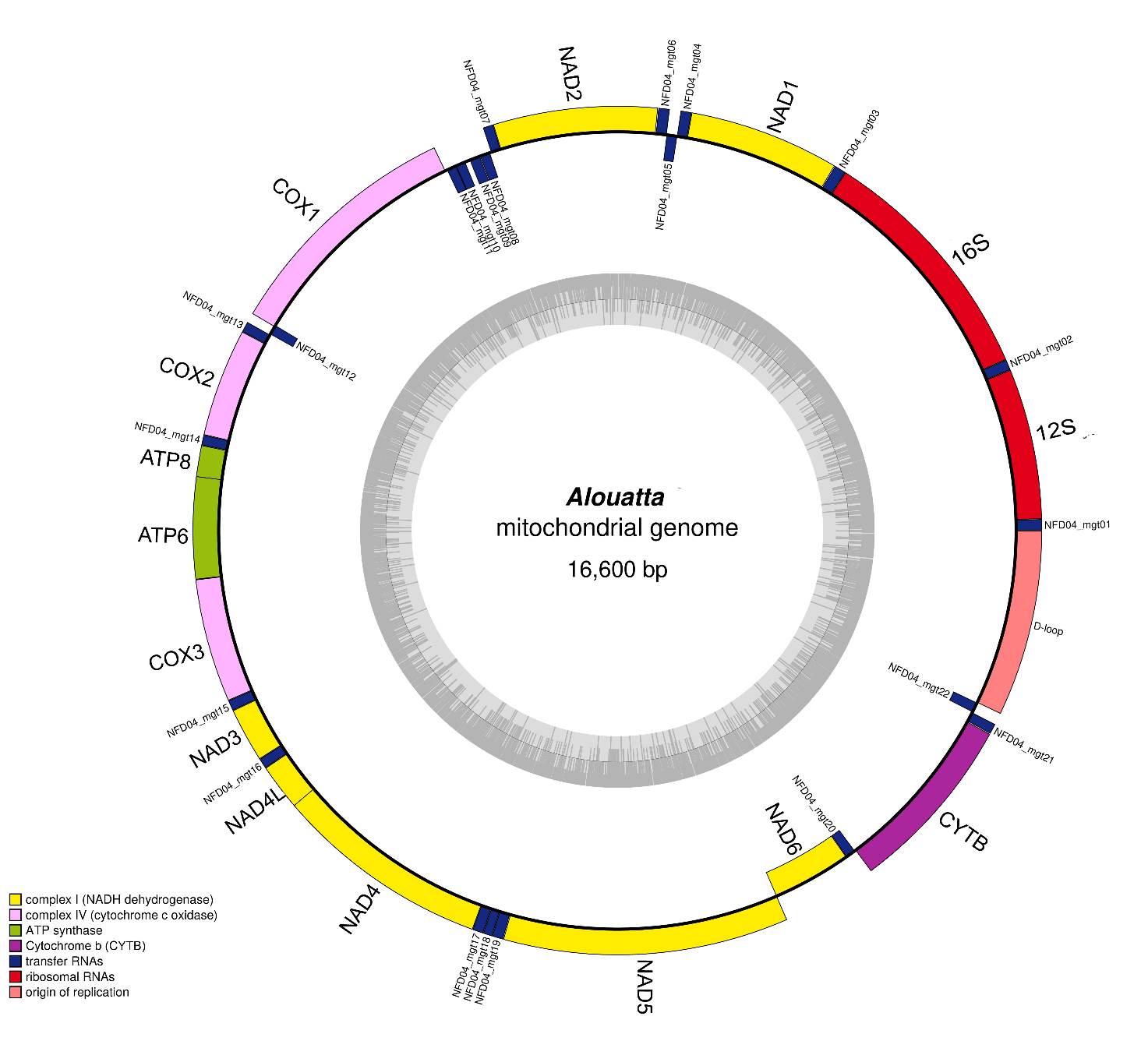


Figure S1. Illustration of the mitochondrial genome generated with OrganellarGenomeDRAW (OGDRAW) version 1.3.1 (Greiner et al., 2019) showing the 37 partitions, emphasizing the 13-protein coding genes.


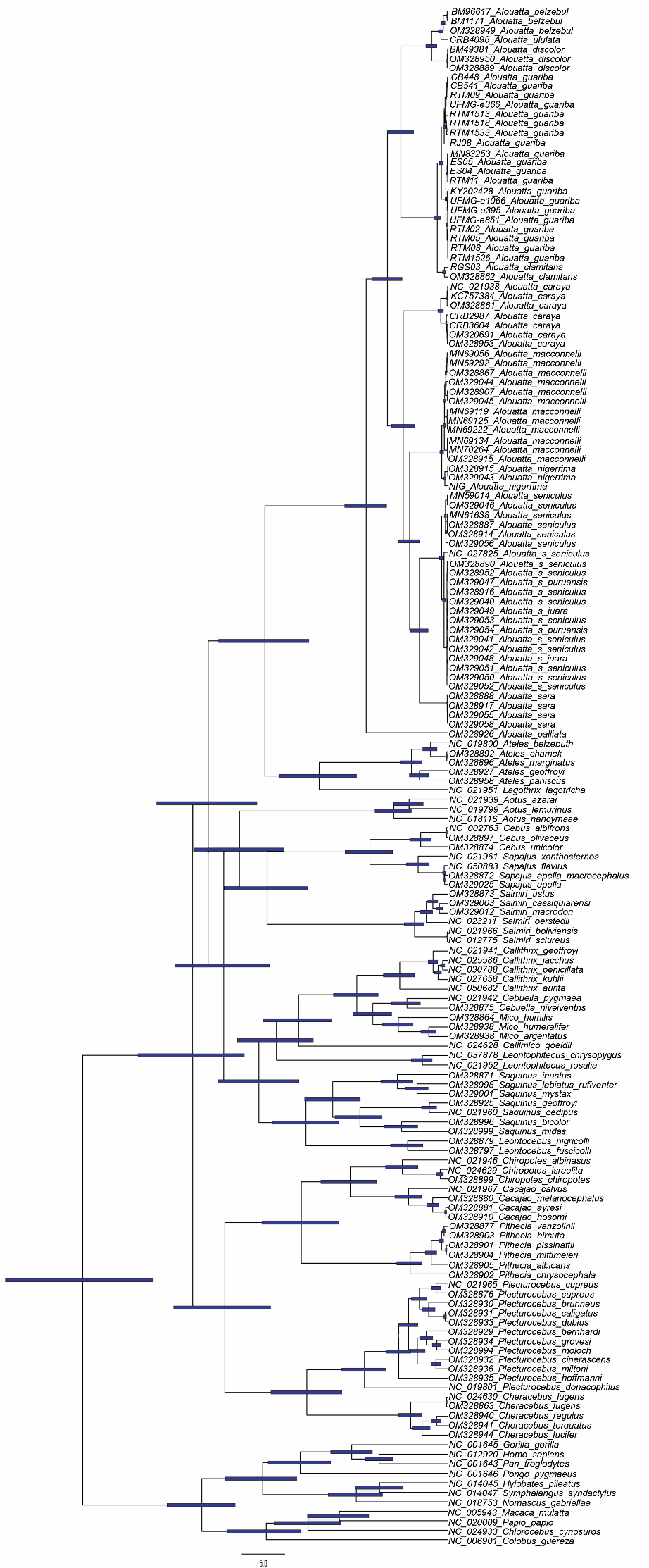


Figure S2. Bayesian time-scaled tree inferred from concatenated mitochondrial coding genes, 12S, and 16S sequences of Anthropoidea focused on the *Alouatta* diversification. Bars indicate 95% highest posterior density (HPD) intervals of the age estimates million years ago (Ma).


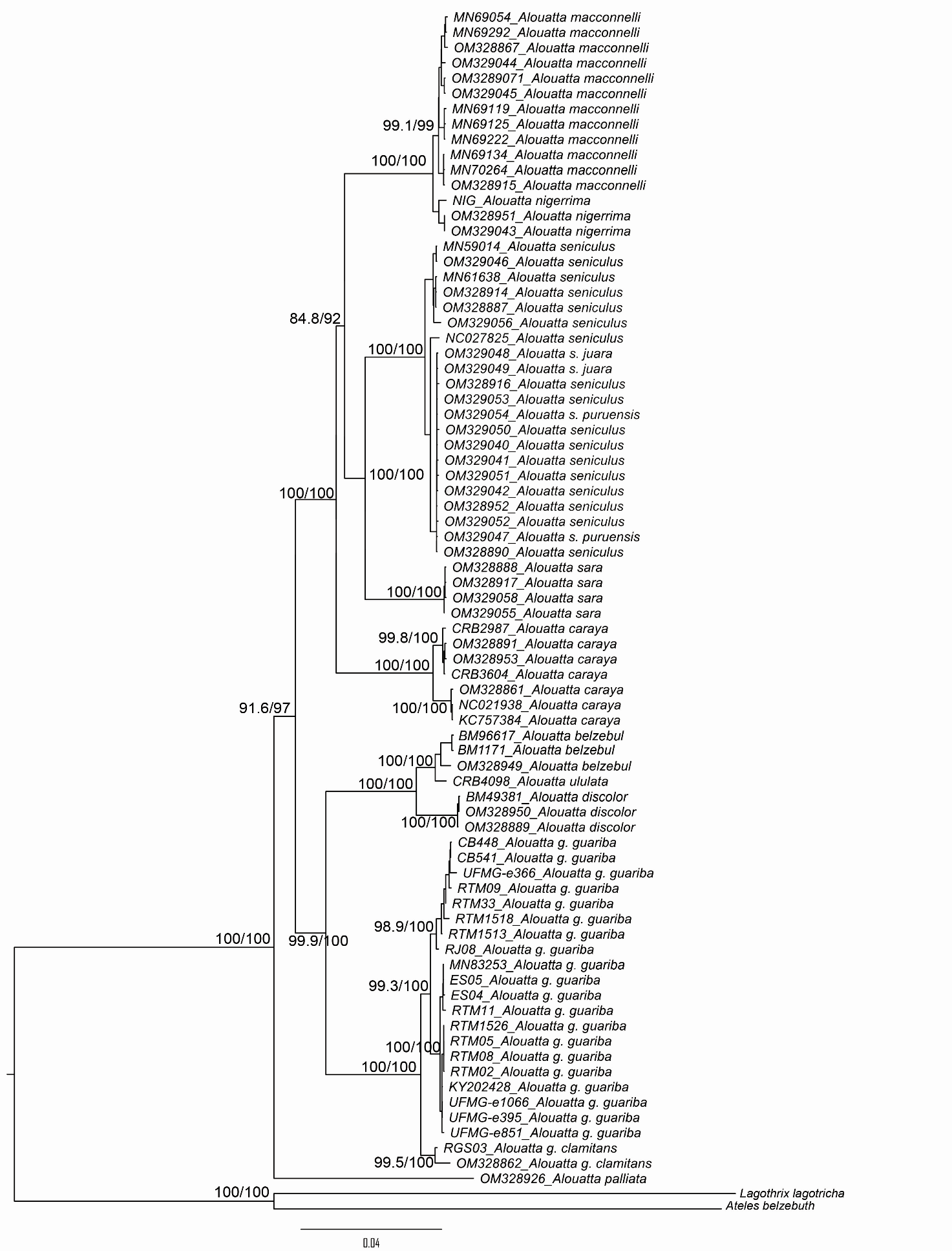


Figure S3. Maximum likelihood tree of *Alouatta* with the concatenated mitochondrial coding genes, 12S, and 16S sequences. The values next to the nodes represent values of SH-LRT and ultra-fast bootstrap, respectively.


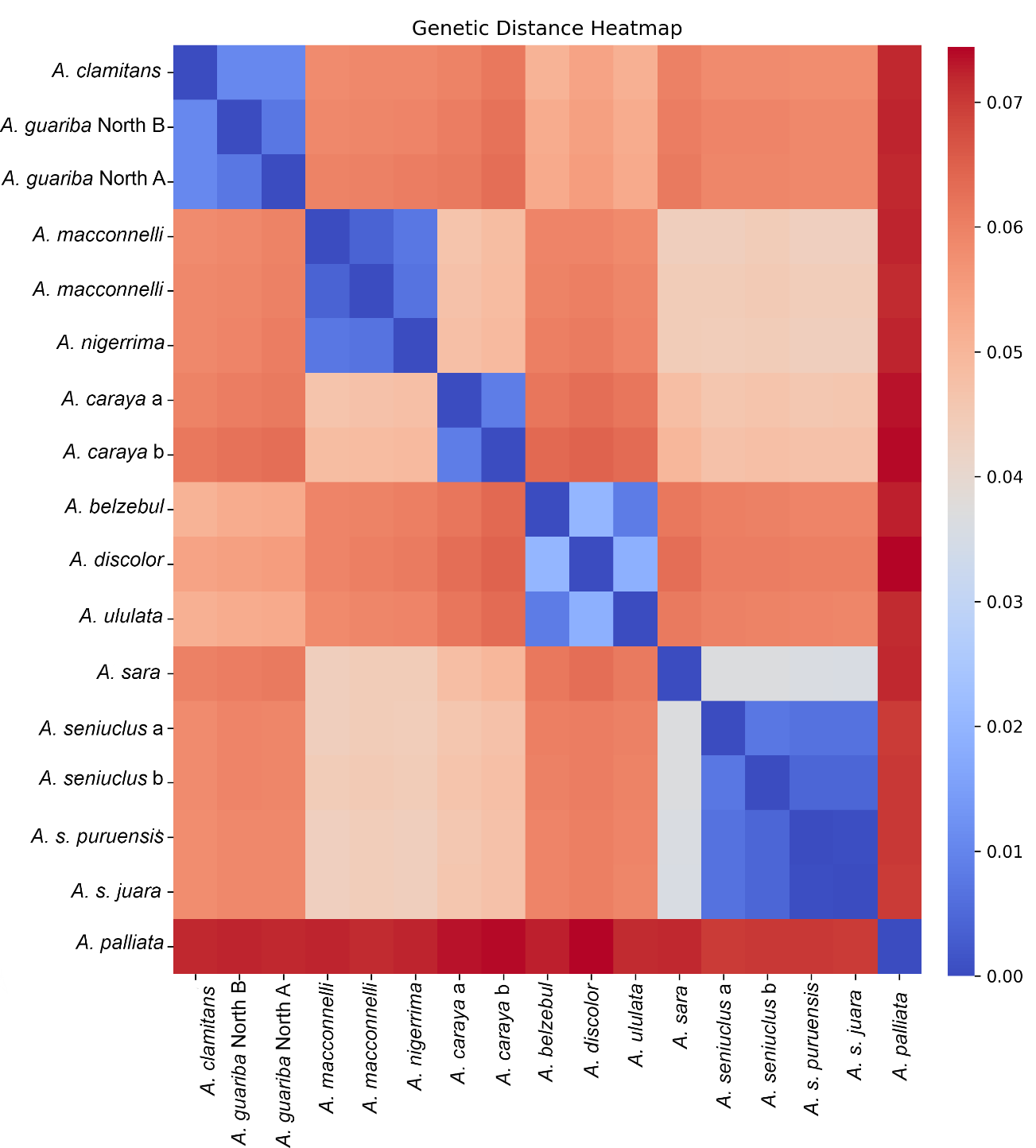


Figure S4. Identity matrix of pairwise genetic distances generated from concatenated mitochondrial coding genes, 12S, and 16S sequences based on the clades of *Alouatta* found in the mitogenome tree.


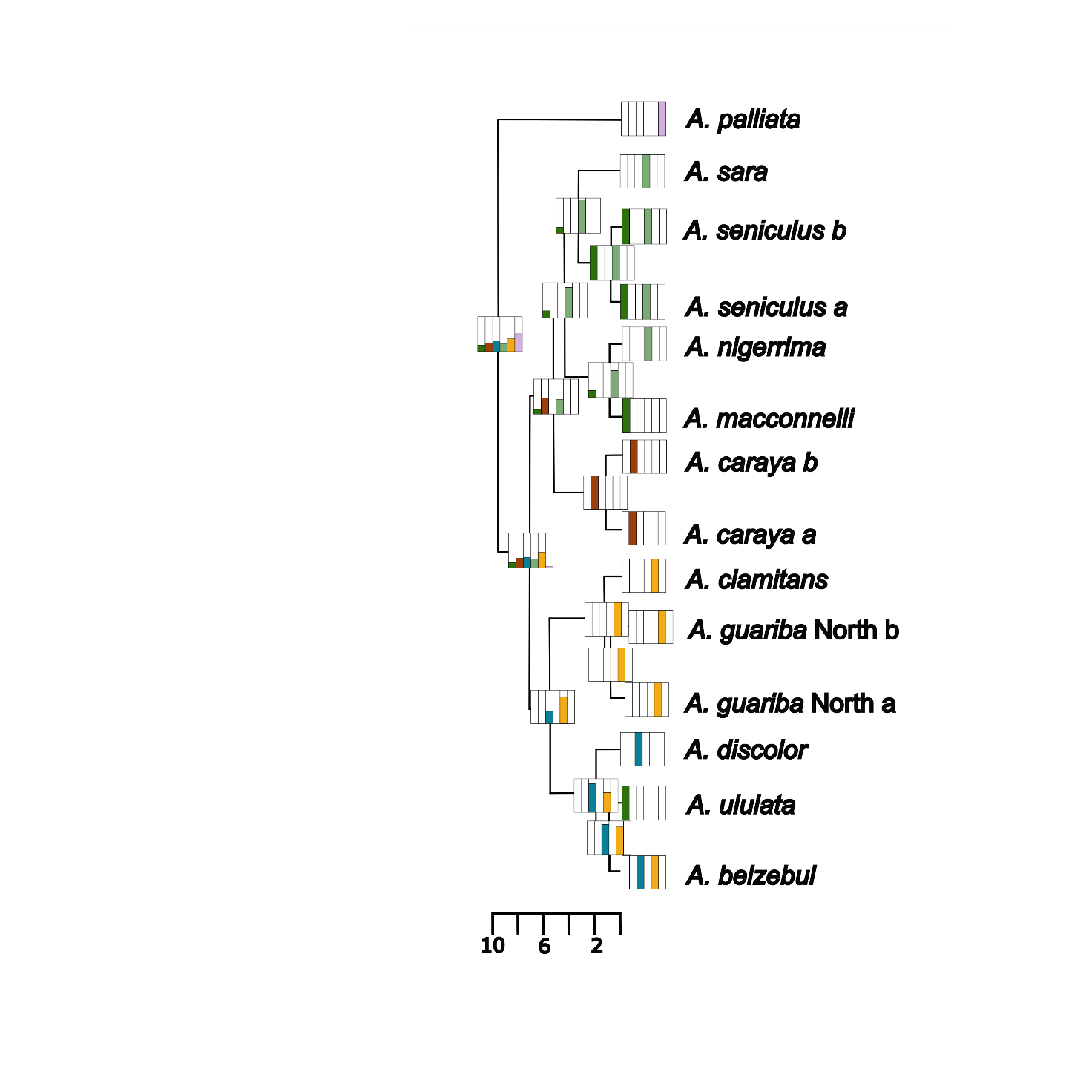


Figure S5. Estimated probability of occupancy per area under the BAYAREALIKE+J model for the ancestors of the *Alouatta* species (internal squares). Terminal squares represent the current distribution of each phylogenetic lineage. Colors represent the regions defined in Figure 4 (main text), southwestern Amazonia (light green), northern Amazon (dark green), south-eastern Amazonia (blue), Atlantic Forest (yellow), and Chaco (brown).

References

Andrews, R. M., Kubacka, I., Chinnery, P. F., Lightowlers, R. N., Turnbull, D. M., & Howell, N. (1999). Reanalysis and revision of the Cambridge reference sequence for human mitochondrial DNA. *Nature genetics*, *23*(2), 147-147.

Arnason, U., Gullberg, A., Burguete, A. S., & Janice, A. (2001). Molecular estimates of primate divergences and new hypotheses for primate dispersal and the origin of modern humans. *Hereditas*, *133*(3), 217-228.

Babb, P. L., Fernandez‐Duque, E., Baiduc, C. A., Gagneux, P., Evans, S., & Schurr, T. G. (2011). mtDNA diversity in Azara's owl monkeys (*Aotus azarai azarai*) of the Argentinean Chaco. *American Journal of Physical Anthropology*, *146*(2), 209-224.

Chan, Y. C., Roos, C., Inoue-Murayama, M., Inoue, E., Shih, C. C., Pei, K. J. C., & Vigilant, L. (2010). Mitochondrial genome sequences effectively reveal the phylogeny of Hylobates gibbons. *PLoS One*, *5*(12), e14419.

Chiou, K. L., Pozzi, L., Alfaro, J. W. L., & Di Fiore, A. (2011). Pleistocene diversification of living squirrel monkeys (*Saimiri* spp.) inferred from complete mitochondrial genome sequences. *Molecular Phylogenetics and Evolution*, *59*(3), 736-745.

de Freitas P. D., Mendez F. L., Chávez-Congrains K., Galetti P. M., Coutinho L. L., Pissinatti A., Bustamante C. D. (2018 ). Next-Generation Sequencing of the Complete Mitochondrial Genome of the Endangered Species Black Lion Tamarin *Leontopithecus chrysopygus* (Primates) and Mitogenomic Phylogeny Focusing on the Callitrichidae Family. *G3 (Bethesda);8*(6), 1985-1991.

Finstermeier, K., Zinner, D., Brameier, M., Meyer, M., Kreuz, E., Hofreiter, M., & Roos, C. (2013). A mitogenomic phylogeny of living primates. *PloS one*, *8*(7), e69504.

Greiner, S., Lehwark, P., & Bock, R. (2019). OrganellarGenomeDRAW (OGDRAW) version 1.3. 1: expanded toolkit for the graphical visualization of organellar genomes. *Nucleic acids research*, *47*(W1), W59-W64.

Hao, Z., & Yi, C. (2019). The complete mitochondrial genome of *Sapajus Flavius* (Blonde Capuchin). *Mitochondrial DNA Part B*, *4*(2), 2970-2971.

Hodgson, J. A., Sterner, K. N., Matthews, L. J., Burrell, A. S., Jani, R. A., Raaum, R. L., ... & Disotell, T. R. (2009). Successive radiations, not stasis, in the South American primate fauna. *Proceedings of the National Academy of Sciences*, *106*(14), 5534-5539.

Horai, S., Hayasaka, K., Kondo, R., Tsugane, K., & Takahata, N. (1995). Recent African origin of modern humans revealed by complete sequences of hominoid mitochondrial DNAs. *Proceedings of the National Academy of Sciences*, *92*(2), 532-536.

Malukiewicz, J., Cartwright, R. A., Curi, N. H., Dergam, J. A., Igayara, C. S., Moreira, S. B., ... & Roos, C. (2021). Mitogenomic phylogeny of *Callithrix* with special focus on human transferred taxa. *BMC genomics*, *22*, 1-14.

Matsudaira, K., & Ishida, T. (2010). Phylogenetic relationships and divergence dates of the whole mitochondrial genome sequences among three gibbon genera. *Molecular Phylogenetics and Evolution*, *55*(2), 454-459.

Matsui, A., Rakotondraparany, F., Munechika, I., Hasegawa, M., & Horai, S. (2009). Molecular phylogeny and evolution of prosimians based on complete sequences of mitochondrial DNAs. *Gene*, *441*(1-2), 53-66.

Menezes, A. N., Viana, M. C., Furtado, C., Schrago, C. G., & Seuanez, H. N. (2013). Positive selection along the evolution of primate mitogenomes. *Mitochondrion*, *13*(6), 846-851.

Raaum, R. L., Sterner, K. N., Noviello, C. M., Stewart, C. B., & Disotell, T. R. (2005). Catarrhine primate divergence dates estimated from complete mitochondrial genomes: concordance with fossil and nuclear DNA evidence. *Journal of human evolution*, *48*(3), 237-257.

Wang, W., Liu, J. Y., Wang, H. F., Yang, M. Y., Liu, Q. Y., & Ding, M. X. (2016). The complete mitochondrial genome of white-tufted-ear marmoset, *Callithrix jacchus* (Primates: Callitrichinae). *Mitochondrial DNA Part A*, *27*(3), 1920-1921.

Zhang, X., Pan, F., & Wu, Z. W. (2016). Complete mitochondrial genome of *Callithrix kuhlii* (Primates: Callitrichinae) with phylogenetic consideration. *Mitochondrial DNA Part A*, *27*(4), 2943-2944.

Zinner, D., Wertheimer, J., Liedigk, R., Groeneveld, L. F., & Roos, C. (2013). Baboon phylogeny as inferred from complete mitochondrial genomes. *American journal of physical anthropology*, *150*(1), 133-140.
